# A Two-Step Prioritization Framework for the Nontargeted LC-HRMS Analysis of Chemicals of Environmental Concern and Byproducts during Drinking Water Production

**DOI:** 10.1021/acsmeasuresciau.6c00061

**Published:** 2026-06-04

**Authors:** Lapo Renai, Giulia Bonaccorso, Federico Sinigaglia, Andrea Ravalli, Daniela Santianni, Massimo Del Bubba

**Affiliations:** † van ‘t Hoff Institute for Molecular Science, 1234 University of Amsterdam, Science Park 904, 1098 XH Amsterdam, The Netherlands; ‡ Department of Chemistry, 9300 University of Florence, Via della Lastruccia 3, 50019 Sesto Fiorentino, Florence, Italy; § 119639 Publiacqua S.p.A., Via di Villamagna 90/c, 50126 Florence, Italy

**Keywords:** liquid chromatography, high-resolution mass spectrometry, online prioritization, design of experiment, offline prioritization, dissolved organic matter, emerging micropollutants

## Abstract

The growing occurrence of chemicals of environmental concern (CECs) in surface and drinking water requires advanced analytical approaches capable of covering the broad chemical space of known and unknown contaminants potentially present in these matrices. Nontargeted analysis (NTA) based on liquid chromatography coupled with high-resolution mass spectrometry (LC-HRMS) is gaining popularity as it is virtually comprehensive and may provide high data quality. However, NTA has some limitations in terms of sensitivity, which may limit its effectiveness, and offline prioritization processes are usually adopted to bring out the desired features from the vast array of NTA signals. A novel LC-HRMS-based two-stage prioritization framework is proposed to evaluate the combined chemical load by dissolved organic matter and CECs in a drinking water treatment plant (DWTP). For the first time, an experimental design (DoE) approach is adopted to optimize LC–HRMS acquisition parameters for the online prioritization of features responsible for the combined chemical load in the investigated samples. DoE was performed on a training set of 42 regulated CECs, characterized by a wide chemical coverage (e.g., log* D* at pH = 7 between −2.49 and 7.38), identifying optimal experimental conditions for maximizing their NTA detection. DoE-optimized online prioritization was combined with offline prioritization, performed using multivariate and univariate analyses, capturing significant trends in dissolved organic matter features, known CECs (e.g., perfluoroalkyl substances), and unknown compounds, tentatively identified as potential pesticide byproducts. Targeted analysis, performed on the CEC training set, provided a 100% match with the NTA approach, thus validating the proposed two-step prioritization framework. The two-step prioritization allows for (i) improving method sensitivity thanks to the online prioritization and (ii) revealing both persistent and potential transformation-derived contaminants, thus providing a comprehensive tool for assessing chemical load and treatment performance in DWTP.

## Graphical abstract

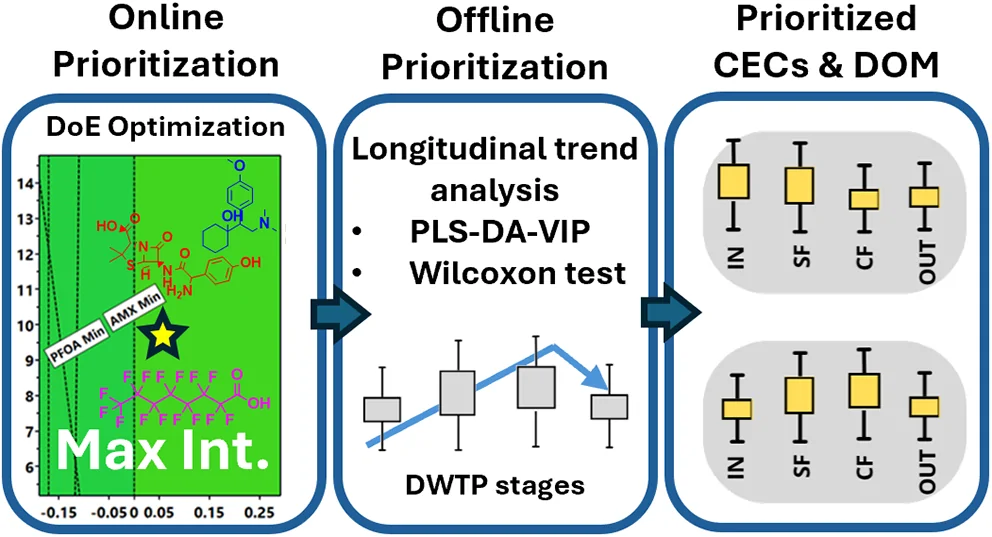

## 1 Introduction

Past and present large-scale human activities are responsible for the current anthropogenic pressure on several environmental compartments and above all on water ecosystems.^1–3^ Such concerning pressure impacts environmental water quality, including aqueous habitats and freshwater resources intended for agriculture and human consumption (i.e., drinking water).^4^ Chemicals of environmental concern (CECs), such as pesticides, antibiotics, surfactants, and personal care products, drive this increasing concern for their ubiquity, physicochemical variability, and potential or unknown risk to ecosystems and human health.^5^ From the punctual or diffuse sources of release (e.g., wastewater treatment plants and industrial/agricultural activities), CECs may degrade the quality of water resources, including drinking water.^6^ Common potabilization steps (filtration and disinfection) involved in drinking water treatment plants (DWTPs) are not designed for the complete removal of CECs. Furthermore, these processes may introduce unknown byproducts that, even at trace levels, pose a potential threat to human health.^7^ Thus, the occurrence of CECs combines with the complex background of dissolved organic matter (DOM), generating a significant “chemical pressure” on DWTP systems that necessitates a comprehensive analytical assessment.

Targeted monitoring by liquid chromatography coupled with mass spectrometry (LC–MS) has been extensively performed for outlining the quantitative balance of specific groups of CECs from DWTP inlet to potable water distribution,^1,8,9^ however being limited by the availability of reference analytical standards and thus neglecting the actual water contamination complexity.^10^

Thanks to the progress made in high-resolution MS (HRMS) platforms, nontargeted analysis (NTA) is gaining popularity due to the superior quality and comprehensive records of the complex chemical space of water samples, tackling the challenges of identifying unknown and/or unexpected CECs and byproducts.^11,12^ The high resolving power (*R* > 20,000) and mass accuracy (<5 ppm), in combination with NTA data processing involving various prioritization strategies, allows for extracting chromatographic and spectral data (i.e., features) of the detected chemical space for known CECs detection (i.e., suspect screening), and also for increasing the possibility to find candidate structures for unknown chemicals (namely, annotation of tentative structures).^13,14^

NTA suspect screening has been extensively employed for the identification of CECs and disinfection byproducts (DBPs) in tap water samples.^15–17^ However, suspect screening allows for capturing only a small portion of the CECs impacting drinking water, since it relies on feature matching against known chemical structures (suspect lists). Furthermore, when suspects belong to particular chemical classes, like for instance DBPs or per- and polyfluoroalkyl substances, their detection is often supported by a specific sample enrichment procedure (e.g., liquid–liquid extraction with MTBE or solid phase extraction), which however impacts the comprehensive measure of sample chemical complexity.^12^

To ensure a greater NTA chemical coverage of water-soluble CECs, it is highly recommended to limit sample manipulation by adopting the large-volume direct injection (LVDI) technique for sample analysis, as well as to establish a prioritization strategy favoring the acquisition of informative chemical features over background noise. Features are prioritized “online” during data acquisition, where data-dependent criteria (e.g., intensity-based selection) determine which precursor ions from the full-scan spectra are targeted for fragmentation. Subsequently, an “offline” prioritization is performed through post hoc data processing; here, features are filtered and ranked based on relative abundance, differential analysis, structural patterns, predicted toxicity, or other quantitative structure–activity relationships (QSAR).^18,19^ Offline prioritization has been widely used in the analysis of drinking water samples, mainly exploiting chemical toxicity prediction models.^20,21^ However, insufficient spectral quality can limit the matching between experimental and in silico MS data. Even though online prioritization strategies could increase the spectral “purity,” they can also heavily reduce the chemical coverage of the acquired data. Consequently, there is a growing need for more balanced acquisition strategies that generate high-quality and informative spectral data while preserving broad chemical coverage.^22^

Efforts are required to prioritize real feature signals during acquisition, tuning the experimental parameters (electrospray and ionization promoters) that can maximize the overall ionization efficiency toward a more comprehensive CEC and CECs-related feature detection and subsequent offline prioritization. Despite this, water analysis workflows remain largely focused on post hoc processing rather than integrating acquisition-driven optimization with offline prioritization, limiting their ability to fully exploit LC–HRMS discovery potential.^23^

In this context, Design of Experiments (DoE) offers a powerful strategy for systematically enhancing instrument performance by jointly varying key LC–MS parameters, such as mobile-phase composition and ESI voltage, and modeling their effects on the analytical response. DoE is widely applied to targeted LC–MS method development to optimize separation for quantitative assessments,^24,25^ yet it finds no application in NTA strategy to maximize the acquisition of high-quality LC-HRMS feature signals during analysis, except for optimizing the parameters of the feature detection algorithm.^26^ If combined with a broad coverage set of analytical standards, DoE key features can assist the online prioritization framework, leading to enhanced ionization efficiency and method performance prior to the analysis of real samples, prioritizing relevant chemical information over background noise during the analysis.

In this work, we present an innovative NTA approach that leverages a two-step online-offline prioritization strategy to assess the presence of known, unknown, and unexpected CECs, as well as DOM, throughout the entire drinking water production process within a DWTP. To this end, we optimized the LC–HRMS acquisition introducing for the first time the synergistic use of DoE strategy for feature online prioritization, to enhance the detection of CEC-related signals by LVDI of water samples. Optimization was guided by maximizing LC–HRMS responses (i.e., absolute peak area) for a training set of 42 regulatory-relevant CECs with diverse physicochemical properties, thus boosting method sensitivity without additional sample manipulation, preserving a wide chemical space coverage of drinking water samples. The overall features detected under the optimized conditions were further prioritized through complementary differential analyses to capture significant DOM trends across the potabilization process, providing a comprehensive picture of the chemical load within the investigated DWTP.

## 2 Materials and Methods

### 2.1 Chemicals and Reagents

Water and methanol (MS grade) used for LC–HRMS analyses were obtained from Honeywell (Milan, Italy). Ammonium formate and formic acid (MS grade, purity ≥99.0%) used for the preparation of the LC eluents were purchased from Sigma-Aldrich. Ultrapure water (resistivity >18 MΩ) was obtained from a Milli-Q system (Millipore, Billerica, MA, USA). Disodium EDTA (purity >99%) was purchased from Phargam s.r.l. (Milan, Italy). The purchase details, abbreviations used, CAS number, major physicochemical properties, and precursor ion features (Table S1) of the 42 target analytes included in the training set are reported in Section S1.1 of the Supporting Information. Minisart regenerated cellulose syringe filters, used for the filtration at 0.2 μm of water samples, were supplied by Sartorius (Gottingen, Germany).

### 2.2 LC–HRMS Analysis

Chromatographic separation was performed on a TriPlus RSH EQuan 850 LC System (Thermo Fisher Scientific, Waltham, MA, USA) in reversed-phase mode, using a PFP Kinetex analytical column (100 mm × 3.0 mm, 2.6 μm), thermostated at 35 °C, and MS-grade water and methanol as elution solvents. The delay column was a Hypersil GOLD (50 mm × 2.1 mm, Thermo Fisher Scientific). Under the optimized conditions, the mobile phase was composed of water (solvent A) and methanol (solvent B) with 0.057% of formic acid and 9.73 mM of ammonium formate. The gradient elution program, expressed as percentage of solvent B in the mobile phase, was as follows: 0–1 min, isocratic 5% B; 1.1–30 min, linear gradient 5–95% B (i.e., about 3.1% per minute); 30.1–31 min, linear gradient 95–5%; and 31–35 min isocratic 5% B. Total run time was 35 min. The mobile-phase flow rate was 0.4 mL/min, and the injection volume was 100 μL. The LC flow was diverted to waste in the beginning (0–2 min) and at the end (30.5–35 min) of the chromatographic gradient. The LC system was coupled to a hybrid linear ion trap Fourier Transform (LTQ FT) Orbitrap high-resolution mass spectrometer, model Exploris 120 (Thermo Fisher, Waltham, MA, USA), by an electrospray ionization (ESI) probe for full-scan MS and data-dependent analysis (DDA) operating in both positive (PI) and negative (NI) ionization. Optimized source-dependent parameters were set as follows: ion transfer tube temperature 290 °C, vaporizer temperature 348.2 °C, sheath gas 30, auxiliary gas 6, and spray voltage 2540 V (for both PI and NI).

Full-scan MS (mass range from 100 to 1000 Da) was performed at a mass resolution of 60,000 FWHM (200 *m*/*z*) in profile mode, RF lens 70%, and an automatic AGC target. DDA experiments were carried out in the 70–1000 *m*/*z* range at a resolution of 30,000 FWHM with an isolation window of 3 Da (MS1 event). Additionally, the following data-dependent criteria were applied for the cycle number of dependent scans (MS^2^ event with TopN = 4 and resolution 15,000 FWHM), increasing the probability of relevant precursor ions to be fragmented as well as to increase MS/MS spectra quality. Intensity threshold was set at 5 × 10^4^ (both NI and PI), isotope filter was set as “assigned”, dynam exclusion was set at one exclusion for 2 s (tolerance of 10 ppm). Full lists of NI- and PI-targeted exclusions are shown in Supporting Information, appendix 1. Targeted inclusion list was implemented as described in Section 2.5.2. Product ions were generated by HCD using stepped collision energies ranging from 30 to 60. AGC target was set to 100 and injection time to 60 ms. Calibration of the instrument was performed prior to analysis in each PI and NI mode using Thermo Pierce FlexMix calibration solution.

PI and NI data sets were acquired in separate randomized batches. Ultrapure water (2 mM EDTA) instrumental blanks and solvent injections (i.e., methanol) were acquired for background subtraction, exclusion list building, and as a control for potential laboratory and instrument contamination. For the analytes belonging to the CEC training set that were detected by the optimized method, all of the samples were reanalyzed in target single-ion monitoring (tSIM) acquisition mode, and external calibration curves were used for quantification.

### 2.3 Samples and Study Design

Sampling campaign was conducted from March to June 2023, and water samples were collected at the inlet of the “Anconella” DWTP (43°45′50.5″N, 11°17′36.5″E), managed by Publiacqua S.p.A (Florence, Italy), and at specific treatment stages as reported with full details in Table S2 of the Supporting Information, thus resulting in a longitudinal sampling strategy. In more detail, the following samples were collected: (i) inlet of the Anconella DWTP, which consists in the water from Arno River after mobile and fixed barriers, and mechanical screens (ANC_IN); (ii) effluent from the sand filters (ANC_SF); (iii) effluent from the granular activated carbon filters (ANC_CF); and (iv) effluent from the Anconella DWTP after the final disinfection stage (ANC_OUT), before the water is pumped into the municipal aqueduct.

A total of 9 samples for each treatment stage over the investigation period of four months were collected. Samples were collected in polyethylene bottles and, after inlet water turbidity analysis, immediately divided into 6 aliquots of 1.5 mL, which were stored at −20 °C until analysis. Pooled samples used as (variability) quality controls (QCs) of each sampling day were obtained by mixing 375 μL of the four sampling points in an Eppendorf tube of 1.5 mL. Before the analysis, samples were thawed at room temperature and filtered into polypropylene vials. All samples and ultrapure water solutions were pretreated by adding an EDTA aqueous solution (2 mM final concentration) after filtration. Subsequently, samples and QCs were acquired under complete, randomized analytical batches to reduce the analytical drift bias, including solvents (i.e., methanol) and blank water injections.^27,28^

### 2.4 Design of Experiment

The DoE optimization workflow focused on maximizing the nontargeted detection (i.e., ionization efficiency) of CECs in aqueous samples by LVDI in the LC–HRMS platform. Ionization performances in the investigated experimental domain were evaluated, considering as “critical method attributes” (CMAs) the chromatographic areas of the physicochemical heterogeneous training set encompassing 42 CECs (Table S1). The “critical method parameters” (CMPs) influencing the selected CMAs in reversed phase chromatography/electrospray ionization (ESI) were identified as follows: (i) eluent acidity—expressed as percentage of formic acid in the mobile phase (FoA), (ii) eluent modifier—expressed as mM concentration of ammonium formate (FoNH4), (iii) source ion spray voltage in kV (IV), and (iv) ion source temperature in °C (IT). CMPs were investigated at 3 levels (see Section S1.3 of the Supporting Information) through a *D-optimal* design (*n* = 3 center points), considering mixed and two-factor interactions. Table S3 shows the design matrix consisting of 23 experiments in total for the experimental domain investigation.

Quadratic modeling (see eq S1) was selected for each CMA for the response surface methodology, and data were fitted using the PLS algorithm (see eqs S2–S5). To increase DoE robustness, data were transformed (e.g., log transformation), and model coefficients were refined (i.e., exclusion of not significant coefficients) when needed. The CMA contour plots inside the experimental domain were finally combined to identify the optimal regions (sweet spots) characterized by the lowest probability of failure (∼1%) and the best log­(D) (i.e., desirability function) in which optimal experimental set points are found. The selection of the optimal set point was based on the alignment between experimental results and model predictions. A ratio of experimental to predicted peak areas equal to 1.0 represents an ideal prediction; thus, a threshold of 0.75 was established as a benchmark for satisfactory predictive accuracy. MODDE 13.0 software (MKS Umetrics AB, Sweden) was employed for DoE planning and the related data treatment toward optimization.

### 2.5 Post Hoc Data Analysis and Annotation Workflow

#### 2.5.1 LC–HRMS Data Preprocessing and Feature Detection

All raw data were visualized and inspected using the FreeStyle software (version 1.8 SP2, Thermo Fisher). LC–HRMS (i.e., full-scan MS) and LC–HRMS/MS (i.e., DDA) files were converted to .*mzXML* and .*mzML*, respectively, via MSConvert provided by the ProteoWizard package.^29^ For both types of acquisition, binary encoding was fixed at 32 bits and the intensity threshold was set to 2 × 10^4^. The scan range was set to 900–13,040 and 1740–26,850 for full-scan and DDA, respectively.

Then, the converted files were processed with the *xcms* open Bioconductor R package that allows for obtaining detection and retention time alignment for nontargeted feature profiling (i.e., ions characterized by a unique exact mass and retention time).^30,31^

Once imported, full-scan NI and PI data underwent peak detection (*findChromPeaks* function) using the *centWave* algorithm with the following inputs: ppm tolerance = 5, peak width 3–60 s, noise = 10,000, and prefilter = 6–5000. Subsequently, neighboring peaks were merged, and alignment was performed using the *obiwarp* method (bin size = 1).^32^ Finally, correspondence and gap filling were carried out to match signals and missing values across samples, as well as blank subtraction to remove undesired features. The obtained NI and PI feature data sets were explored by means of principal component analysis (PCA, *factoextra* package) to detect any batch effect affecting the samples. To this extent, a QC-based center scaling of both data sets was performed to remove signal drifts.

In parallel, analytical batch blanks were processed to extract background and noisy features to perform the blank subtraction on NI and PI full-scan data and to build a targeted exclusion list for DDA experiments. Inputs for the *centWave* peak detection were modified to detect broader chromatographic “features” occurring within blanks.

#### 2.5.2 Offline Feature Prioritization

The features inside the NI and PI data sets were offline prioritized using two parallel strategies. A first prioritization was performed using partial least-squares regression-discriminant analysis method (PLS-DA), extracting the significant features by their variable importance projections (VIPs).^33^ To this extent, the *mdatools* package and the *plsda* function were used.^34^ VIP scores were extracted, and the features were prioritized, retaining those characterized by a VIP score of >1 in at least one of the treatment samples.

An additional prioritization strategy was carried out through a post hoc test using *stats* and *rstatix* packages. In detail, after NI and PI features were tested for homogeneous variance and normality (Flinger and Shapiro tests, respectively), the pairwise Wilcoxon test was carried out, performing multiple comparisons and *P*-value adjustment (Benjamini-Hochberg method). NI and PI significant features (*p*-values <0.05) were retained. The prioritized feature lists for both NI and PI data sets were used as a targeted inclusion list for DDA experiments.

#### 2.5.3 Feature Annotation

Annotation was performed according to the pertinent levels of identification confidence reported in Table 1.^35^ Feature structure was confirmed by means of a reference standard matching with MS, MS/MS, and retention time (level 1). To this extent, we recorded DDA data of the 42 CEC training set (see Section 2.1). In addition, prioritized features with no information for formula assignment (level 5) were considered relevant to DOM assessment. Once converted in .*mzML*, LC–HRMS/MS files were processed for annotating unknown features beyond level 1. A first step involved the comparison of experimental MS^2^ data (*compareSpectra*) with library records extracted from GNPS repository,^36^ as to achieve level 2 annotation. In addition, level 3 annotation was carried out by *MetFragR* package, allowing for PubChem database candidate structures (5 ppm tolerance) to be fragmented *in silico* and matched against experimental fragment *m*/*z* values (50 ppm tolerance).^37^

**Table 1:** Annotation Confidence Levels Adopted in This Study^a^

annotation levels	description	confidence
Level 1	Confirmed structure by reference standard with MS, MS/MS, t_R_ matching.	High
Level 2	Probable structure matching library or literature MS data, or unique diagnostic evidence	Medium
Level 3	Tentative candidate(s) matching MS and (*in silico*) MS/MS, but insufficient information for one exact structure.	Medium-Low
Level 4	Unequivocal molecular formula assigned using the spectral information	Low
Level 5	Measurable exact mass, no formula assigned.	Low

a MS: precursor m/z value; MS/MS: tandem mass spectrum/fragmentation pattern; t_R_: retention time.

### 2.6 Calculation and Data Availability

Data processing and statistical analyses were performed using R (version 4.4.2). LC–HRMS. Code for data processing, offline prioritization, and annotation are available in the Supporting Information file “Code_markdown.pdf”.

## 3 Results and Discussion

Figure 1 provides a schematic overview of the study design, illustrating the (online prioritization) LC–HRMS optimization, data acquisition, offline prioritization, and annotation steps of the NTA workflow.

**Figure 1:**
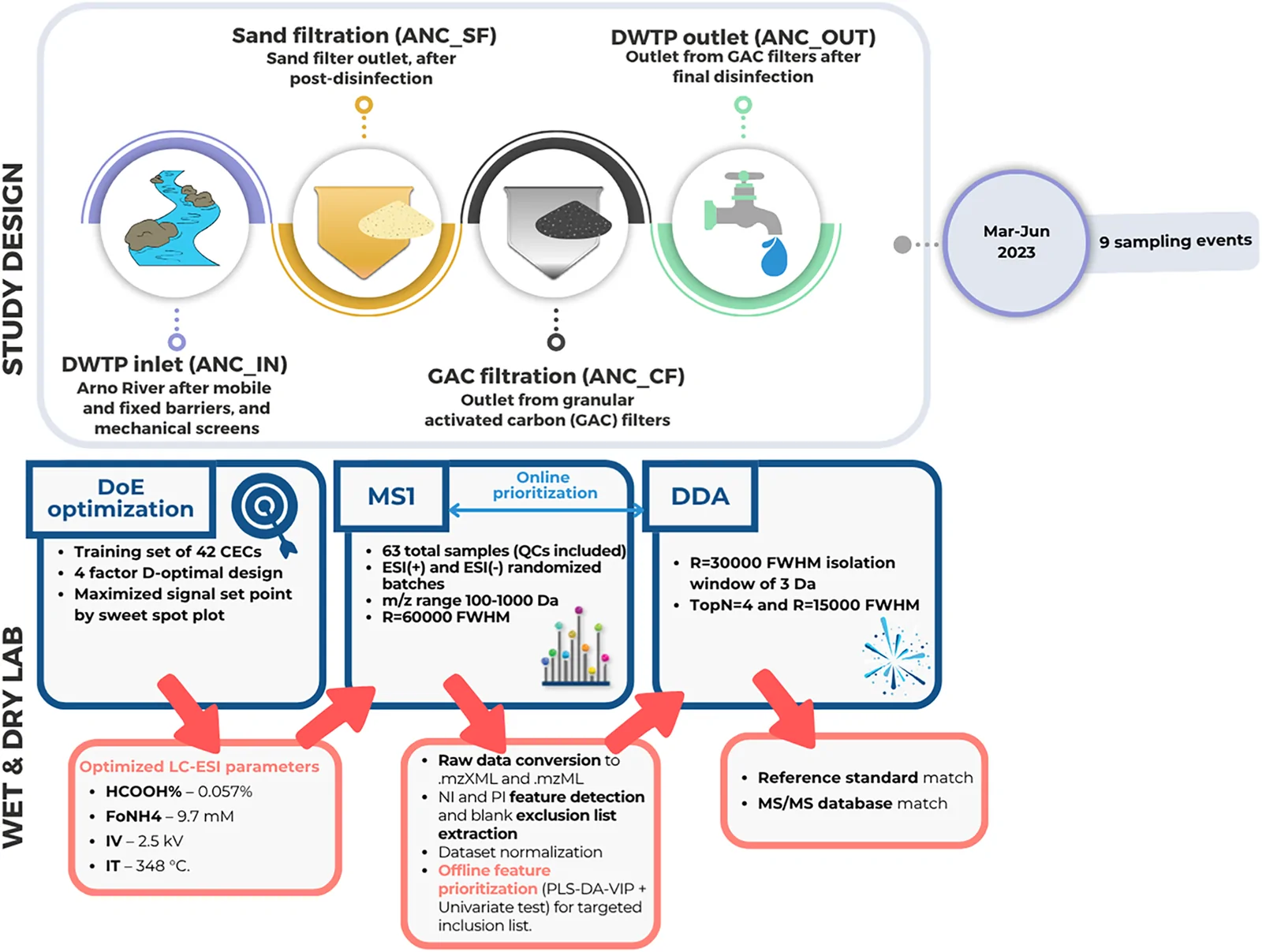
Schematized study design and NTA strategy, including DoE optimization for online prioritized acquisition, feature (offline) prioritization, and annotation steps.

For each sampling event, seven water samples (including *n* = 3 QC replicates) were analyzed, resulting in a total of 63 samples. Sampling along the drinking water supply chain was triggered by periods of elevated inlet water turbidity (>1 NTU) and total organic carbon (TOC > 1 mg/L), which served as indicators of increased DOM entering the treatment plant. DOM comprises a diverse and heterogeneous mixture of substances derived from natural biological processes in aquatic systems as well as potential anthropogenic inputs, including CECs.^38^

### 3.1 Online Prioritization by DoE

#### 3.1.1 DoE Optimization

The DoE approach was exploited to promote the detection of CEC-related features by LVDI of water samples as part of the online prioritization strategy. PLS statistics (i.e., ANOVA) performed on the CEC training set yielded significant models in regression and/or validation regression and/or validation for 18 target analytes out of 42 (Table S4 of the Supporting Information), which were retained as characterized by at least *R* ^2^ > 0.5, *Q* ^2^ > 0.1, validity >0.25, and reproducibility >0.5, according to the MODDE guidelines. The retained PFASs, PFDoDA-excluded, were interestingly characterized by very high *R* ^2^ and *Q* ^2^ values, similar to what was observed for several PhCs (i.e., CIP, CLA, DIC, E2, and E1).

The graphical analysis of the refined PLS coefficients reported in Figure S1 allows for highlighting the role of the linear, quadratic, and interaction terms of each CMP toward the maximization of each retained CMA. Remarkably, among the selected CMPs, FoA emerged as a dominant factor driving the ionization efficiency across the CEC training set. For example, the presence of FoA in the eluent neutralizes the charge of PFAS, while it can enhance the response of basic compounds through their protonation and charge acquisition. This result suggests the importance of carefully selecting the FoA concentration in the eluent to find the optimal compromise regarding sensitivity. In Section S1.3 of the Supporting Information, a more detailed comment is reported regarding the trend of the PLS coefficients.

Figure 2 shows the sweet-spot plot obtained by the response surfaces of the considered 18 CMAs meeting the maximization criteria. The identification of this optimal experimental region was supported by specific design coordinates (namely experiments N10 and N20), which demonstrated the highest cumulative maximization of the selected CMAs (Figure S2 of the Supporting Information). Inside this experimental area, three set points were identified with the lowest probability of failure (≤0.5) and the best log­(D) (0.8–0.9) as potential online feature prioritization conditions. Table S5 shows the results of the comparative ratio between experimental and sweet-spot plot predicted chromatographic areas of the 18 CMAs, to test the robustness and reliability of each set point. Based on this assessment, the highest frequency of experimental/prediction ratio >0.75 (*n* = 11), and broadest chemical coverage (bold ratios in Table S5) were obtained in the following experimental conditions: FoA – 0.057%, FoNH4 – 9.7 mM, IV – 2.5 kV, and IT – 348 °C. This optimized setup was applied to the analysis of the selected water samples to prioritize the ionization and subsequent detection of known CECs and CEC-related soluble chemicals.

**Figure 2:**
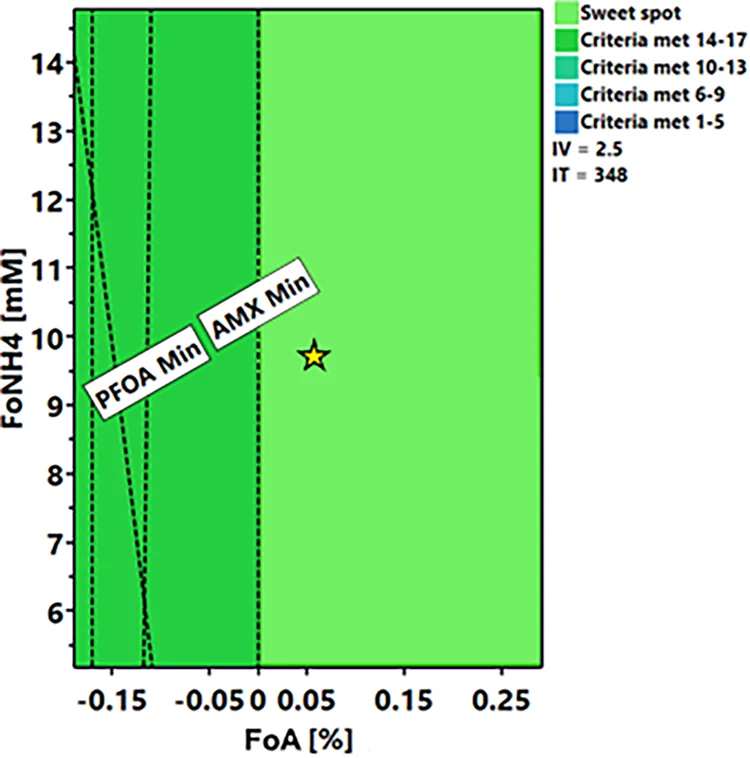
Sweet-spot plot of FoA% vs FoNH4 (IV = 2.5, IT = 348 °C) for the 18 significant CMAs, targeting chromatographic area maximization. The star point refers to the best compromise in terms of chromatographic area for the selected CMAs, i.e., the optimal set point (HCOOH% = 0.057%, FoNH4 = 9.7 mM).

#### 3.1.2 Annotated Features by Online Prioritization

To demonstrate the effectiveness of the optimized online prioritization performed by DoE, level 1 matches with the CEC training set were checked throughout the NI and PI data sets. Within this framework, 3 PFASs were annotated, namely PFBS (FT0963), PFHpA (FT1287), PFOA (FT1499), and O-VEN (FT1483), which matched with the reference standards by retention time, exact mass (Δ<5 ppm), and fragmentation pattern (i.e., level 1), as reported in Table 2.

**Table 2:** List of the Level 1 Annotated Features in the Water Samples^a^

ID	*t*_R_	adduct	*m*/*z*	formula	Δ	MS/MS	annotation	InChiKey
FT1483	10.6	[M + H]^+^	264.1956	C16H25NO2	–0.7	246.7847; 201.1270:133.0646; 107.0489	O-VEN	KYYIDSXMWOZKMP-UHFFFAOYSA-N
FT0963	16.4	[M – H]^−^	298.9438	C4HF9O3S	3	168.9891; 118.9921; 79.9573	PFBS	JGTNAGYHADQMCM-UHFFFAOYSA-N
FT1287	20.2	[M – H]^−^	362.9710	C7HF13O2	3.9	318.9798; 168.9891; 118.9924	PFHpA	ZWBAMYVPMDSJGQ-UHFFFAOYSA-N
FT1499	22	[M – H]^−^	412.968	C8HF15O2	1.2	368.9757; 218.9860; 168.9891	PFOA	SNGREZUHAYWORS-UHFFFAOYSA-N

a Feature identifier (ID), retention time (*t*_R_, min), MS adduct, experimental mass in full-scan spectrum (m/z, Da), candidate formula, mass accuracy (Δ, ppm), main MS/MS fragments, feature annotation, and InChiKey.

Figure S3 of the Supporting Information shows the features’ box-whiskers plots along with the sampling points, which were characterized by an overall low-intensity profile at each stage of the potabilization process, with intensities near the method’s feature detection limit (i.e., 1 × 10^5^). However, the occurrence of high transient signals (i.e., boxplot outliers) related to specific sampling events suggests a key evidence justifying the necessity of a temporal sampling strategy to isolate the trace-level trends of these CECs from the complex chemical background of DOM; a single, nonreplicated sampling event would likely have failed to capture the presence of these contaminants, leading to an underestimation of the “chemical pressure” exerted on the DWTP. In more detail, even though PFBS (Figure S3A) decreases from inlet to outlet, showing a remarkable removal trend, PFHpA, PFOA, and O-VEN (Figure S3B–D) exhibit a constant occurrence profile with no effective removal along the drinking water production chain. The output for PFHpA and PFOA may be ascribable to the synergic combination of different sample-dependent contributions, such as a continuous PFAS income, desorption of earlier adsorbed PFASs (in both sand and activated carbon filtration steps), PFAS leaching from treatment materials, and their well-known high recalcitrance to oxidation processes.^39,40^ Interestingly, targeted quantitative analysis carried out on the 42 molecules of the training set detected PFBS, PFHpA, PFOA, and O-VEN, thus confirming the successful online optimization of the acquisition approach proposed here. Although the trends along the DWTP sampling points determined for these features by the targeted analysis (Figure S4 of the Supporting Information) were only partially in agreement with the intensities determined by NTA in the corresponding samples, these differences are somehow expected as they reflect the intrinsic different acquisition characteristics (i.e., tSIM vs full-scan) and analytical sensitivities of targeted and nontargeted assessments. Nevertheless, the trace level concentration (i.e., ng/L range) of the four identified analytes of the CEC training set confirmed by quantitative analysis reflects the proximity to the detection limit for the NTA profiles of these features. Therefore, the low-intensity, nontargeted signals were not due to weak or reduced ionization but were instead caused by the very low concentration of the analytes monitored along the sampling points. This outcome supports the optimal sensitivity achieved by the DoE-driven ionization efficiency optimization in prioritizing the detection of regulatory-relevant CECs, tracking their chemical trends, even in the presence of varying DOM.

### 3.2 NTA Post Hoc Workflow Results

The raw NI and PI data sets originally contained 52 k and 279 k features among the seven samples, respectively. After the feature detection, alignment, correspondence + gap filling, and blank subtraction steps, these numbers were significantly reduced to 1948 features in NI and 4359 in PI.

The trend of the total number of DOM features for each sampling point in the NI and PI data sets is reported in Figure S5A,B, respectively. Both data sets indicate a decrease in feature abundance from the DWTP input (ANC_IN) to output (ANC_OUT), pointing out a general removal during the water treatment process. However, to identify more significant contributions to these trends, such preprocessed and normalized data sets underwent offline prioritization as reported in Section 2.5.2.

#### 3.2.1 Offline Feature Prioritization in Water Samples

Table S6 of the Supporting Information reports the features prioritized by adopting the PLS-DA-VIP strategy and the univariate Wilcoxon test for the differential analysis of features in both NI and PI data sets. A total of 77 and 56 features resulted in significant differences in NI and PI, respectively. The two offline prioritization strategies provided complementary results with no overlap in terms of common features identified by the PLS-DA-VIP and Wilcoxon test approaches, thus highlighting the importance of using both post hoc prioritization protocols.^23,41^ The prioritized features span a precursor mass range of 200–1000 Da and elute across the majority of the chromatographic gradient, showing a good *m*/*z*-*t*_R_ overlap (55 and 73% for PI and NI, respectively) with the physicochemical space of the CECs included in the training set (Figure S6 of the Supporting Information). This distribution suggests that the framework successfully prioritizes a broad range of DOM features physicochemically related to the selected model CECs, consistent with the expected chemical load in the DWTP.

Figure 3 shows four representative feature trends along with longitudinal sampling points for each prioritization strategy (boxes A-D and E-H for PLS-DA-VIP and Wilcoxon post hoc test, respectively) in NI and PI data sets. The PLS-DA-VIP approach explained most of the feature space variance with the first two components (99.8 and 52.4% for NI and PI, respectively), mainly retaining features characterized by evident monotone descending trends (Figure 3A–C) along with the sampling points, with very few exceptions, such as the feature FT3326 (Table S6), whose trend is shown in Figure 3D. A more “punctual” scenario was depicted by the features prioritized by the Wilcoxon post hoc test, as it compares sample medians, assigning ranks to the data to determine whether observed differences are statistically significant. Although similar trends to those observed for PLS-DA-VIP were highlighted (Figure 3E–F), the univariate strategy also prioritized features with a high variability in intermediate sampling points, such as after primary disinfection, flocculation, and sand filtration (i.e., ANC_SF, Figure 3G–H). This may be due to the formation of undesired byproducts (e.g., DBPs),^16,38^ which are however removed in subsequent treatment stages, before the water is distributed into the municipal aqueduct. This demonstrates that drinking water treatment is not chemically monotonic but temporally reactive (e.g., transient chemical pulses highlighted by boxplot outliers), with some compounds formed or released during the various treatment stages before being partially or completely removed in the subsequent treatment phases. The DWTP therefore behaves as a “chemically active reactor” rather than a simple filter, emphasizing the significance of the online and offline prioritization design in a comprehensive NTA study design investigating longitudinal data to evaluate DWTP chemical load. Such a strategy is essential for accurately estimating the total load of chemicals entering the plant or generated during the various stages of the water treatment process. Additionally, it is crucial for understanding the complete or partial removal of these chemicals at the outlet before they enter the distribution network.

**Figure 3:**
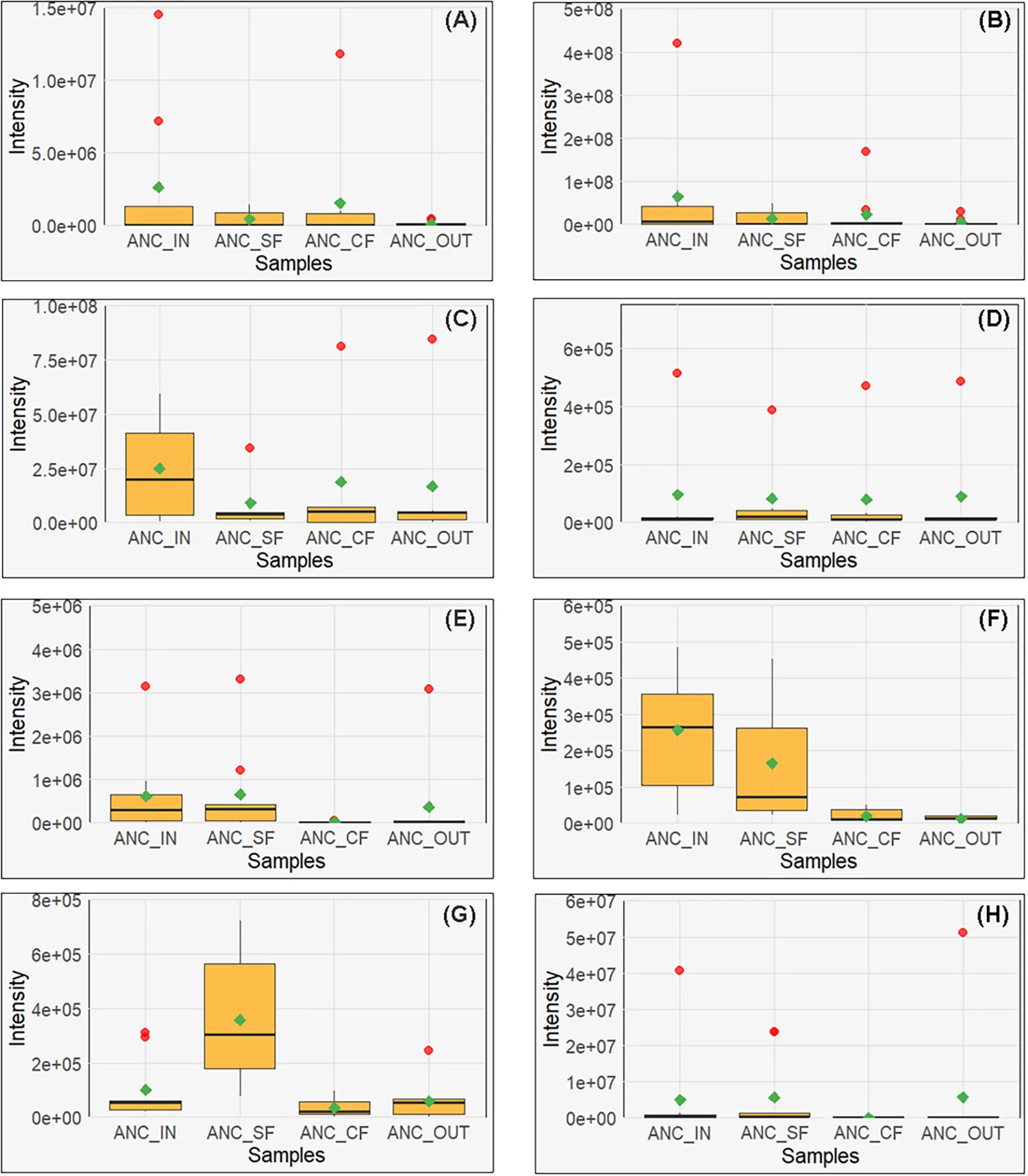
Box-whiskers intensity plots of representative features prioritized by PLS-DA-VIP ((A, B) FT1329-FT2007 in negative ionization and (C, D) FT1699-FT3326 in positive ionization) and the Wilcoxon post hoc test (EF FT0417-FT1051 in negative ionization and G, H FT4039-FT2930 in positive ionization) along the investigated sampling points: ANC_IN (inlet of the Anconella drinking water treatment plant), ANC_SF (effluent from the sand filtration stage), ANC_CF (effluent from the activated carbon filtration stage), and ANC_OUT (outlet of the Anconella drinking water treatment plant). The selected features are shown in bold in Table S6. The yellow boxes represent the interquartile range (sample size *n* = 9), whereas the bold gray line is the median; lower and upper whiskers extend to the maximum or minimum data point within 1.5 box height from the top or bottom of the box; green rhombus is the mean; and full red dots are the outliers representing feature transient fluctuations.

#### 3.2.2 Tentative Annotation of Features in the Drinking Water Supply Chain

Table 3 reports the features annotated according to the procedures and confidence levels reported in Section 2.5.3. Level 2 annotations were neither achieved among prioritized features nor in the entire NI and PI data sets. The tentative compound structures of level 3 annotations, along with longitudinal box-and-whisker plots, fragmentation spectra, and structural elucidation, are reported in Figure 4.

**Figure 4:**
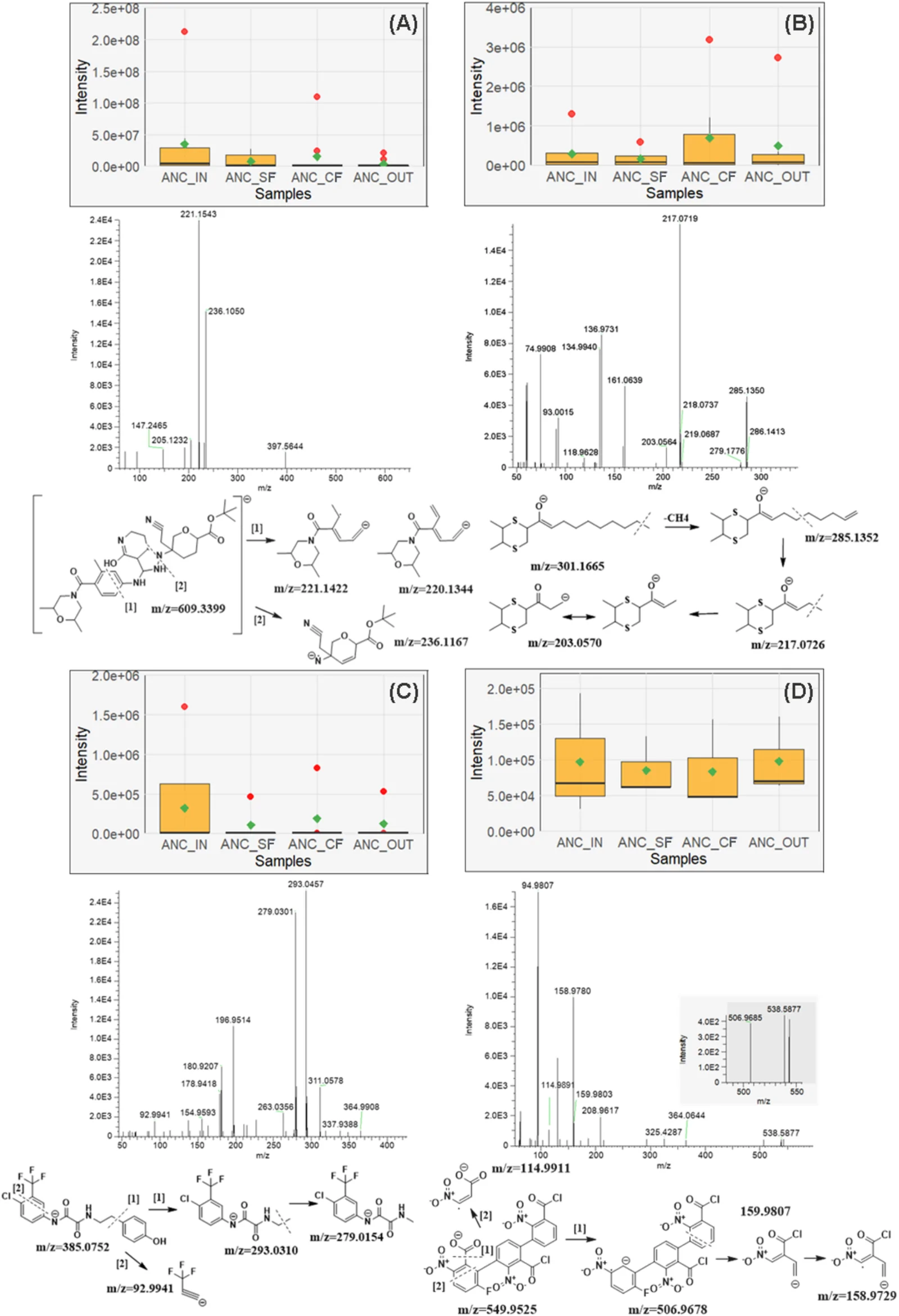
Box-whiskers plots, average MS/MS spectrum, and structural elucidation schemes of the level 2 annotated features (see Table 1) in the investigated water samples. (A) FT2076, (B) FT0974, (C) FT1382, and (D) FT502. The yellow boxes represent the interquartile range (sample size *n* = 9), whereas the bold gray line is the median; lower and upper whiskers extend to the maximum or minimum data point within 1.5 box height from the top or bottom of the box; green rhombus is the mean; and full red dots are the outliers representing feature transient fluctuations.

**Table 3:** List of the Tentative Annotated Features in the Investigated Water Samples^a^

ID	*t*_R_	adduct	*m*/*z*	formula	Δ	MS/MS	annotation	DB	InChiKey	level
Prioritized Features										
FT2076	21.4	[M – H]^−^	609.341	C32H46N6O6	1.1	236.1050; 221.1543; 220.1460	ter-butyl (2*S*,5*S*)-5-(cyanomethyl)-5-[3-[4-(2,6-dimethylmorpholine-4-carbonyl)-3-methylanilino]-4-oxo-3,3a,5,6,7,7a-hexahydro-2H-pyrazolo[4,3-*c*]pyridin-1-yl]oxane-2-carboxylate	146012092	RXCNSIGECUMQJA-NNKGDTHTSA-N	3
FT0974	25.0	[M – H]^−^	301.1662	C16H30OS2	–0.9	285.1352; 217.0719; 203.0565	1-(5,6-dimethyl-1,4-dithian-2-yl)decan-1-one	103344744	XUUGCORWNDCFKF-UHFFFAOYSA-N	3
Other Features										
FT1382	14.7	[M – H]^−^	385.0573	C17H14ClF3N2O3	2.9	311.0578; 294.0484; 293.0457; 280.0319; 279.0301; 92.9941	N’-[4-chloro-3-(trifluoromethyl)phenyl]-N-[2-(4-hydroxyphenyl)ethyl]oxamide	108503033	JPUULLRTBRJYOQ-UHFFFAOYSA-N	3
FT502	30.5	[M + H]^+^	549.9525	C21H8Cl2FN3O10	4.9	506.9685; 159.9803; 158.9785; 114.9891	2-[3-carbonochloridoyl-4-(3-carbonochloridoyl-2-nitrophenyl)-2-nitrophenyl]-3-fluoro-6-nitrobenzoic acid	141902964	BRJVMIMJDGDHGF-UHFFFAOYSA-N	3

a Retention time (*t*_R_, min), MS adduct, experimental mass in full-scan spectrum (m/z, Da), candidate formula, mass accuracy (Δ, ppm), main MS/MS fragments, probable structure annotation, PubChem database identifier (DB), InChiKey, and annotation level.

Among the prioritized features, FT2076 tentative structure was annotated as ter-butyl (2*S*,5*S*)-5-(cyanomethyl)-5-[3-[4-(2,6-dimethylmorpholine-4-carbonyl)-3-methylanilino]-4-oxo-3,3a,5,6,7,7a-hexahydro-2*H*-pyrazolo­[4,3-*c*]­pyridin-1-yl]­oxane-2-carboxylate. Interestingly, such feature was characterized by an almost complete removal from inlet to outlet (Figure 4A), whereas its probable structure shared molecular scaffolds with phenmediphan polar pesticide already detected in surface waters.^42^ Another level 3 annotation was achieved for the prioritized feature FT0974. The prioritized substance shows a consistent fragmentation pattern with 1-(5,6-dimethyl-1,4-dithian-2-yl)­decan-1-one, which shares a molecular structure common in dithiane insecticides.^43^ This finding is characterized by a moderate increase in ANC_CT followed by a decrease in ANC_OUT (Figure 4B), likely attributable to the final disinfection process.

Outside the list of prioritized features, two further compounds were tentatively annotated, namely FT 1382 (NI data set) and FT502 (PI data set). FT 1382 (tentative annotation as *N*′-[4-chloro-3-(trifluoromethyl)­phenyl]-*N*-[2-(4-hydroxyphenyl)­ethyl]­oxamide) was characterized by a remarkable removal trend during treatments (Figure 4C), and its structural elucidation confirmed the occurrence of molecular scaffolds related to polar pesticide substructure (e.g., tau-flavulinate and fluopyram).^42,44^ FT502 was annotated at level 3 as 2-[3-carbonochloridoyl-4-(3-carbonochloridoyl-2-nitrophenyl)-2-nitrophenyl]-3-fluoro-6-nitrobenzoic acid (Figure 4D). Its constant occurrence along the sampling points evidences ineffective removal by the DWTP stages; however, it is characterized by an average intensity around the method limit of feature detection. A recent study evidenced the occurrence of nitro compounds originating from oxidized transformation products of precursor amine moieties, confirming the significant number of nitro groups in the annotated compound.^45^

Although we are aware of the discrepancy between the number of prioritized and annotated features, the framework proposed here prioritizes a broad chemical coverage. This trade-off between selectivity and sensitivity by a two-step prioritization framework has been conceptualized to holistically capture the often underestimated DOM background within the drinking water production chain, beyond pursuing feature annotation only.

## 4 Conclusions

This study presents a comprehensive and data-driven nontargeted analysis workflow to evaluate chemical load during various stages of the drinking water production process. By integrating a DoE approach for online prioritization with multivariate and univariate offline prioritization strategies, the proposed workflow enables a holistic interpretation of DOM trends and CEC occurrence across different treatment stages within a complex potabilization facility treating river water.

DoE-optimized online prioritization has proven to be effective in improving the performance of the LC–HRMS method, enabling the acquisition of a large number of CEC-related features via LVDI, without extensive sample pretreatment, and preserving the native chemical profile of the water samples.

The subsequent application of offline prioritization allowed the identification of statistically significant trends in DOM along the treatment chain. The combined use of multivariate (PLS-DA) and univariate (Wilcoxon) analyses revealed significant DOM trends, offering valuable insights into the DWTP chemical load; in fact, DWTPs act as dynamic chemical reactors in which contaminants can be removed, generated, and released. CECs belonging to PFAS and pharmaceuticals were successfully identified, alongside unknown features tentatively linked to suspected degradation or byproduct formation. While the observed longitudinal trends are consistent with shifts in the chemical load during treatment, the structural identification of tentative byproduct formation must be considered indicative rather than confirmatory. Nonetheless, the recorded tentative and unidentified chemical information constitutes a valuable digital resource, providing a realistic baseline for the future tracking and retrospective reanalysis of chemical load within the DWTP, also impacting human exposure.

## Supplementary Material







## Notes

Code markdown (PDF)
Extended methodological details (sections S1 and S2), including figures (S1–S6), and tables (S1–S6) that support the prioritization framework and real sample analysis (PDF)
Appendix 1 (XLSX)
