# Supplementary material for: A Two-Step Prioritization Framework for the Nontargeted LC-HRMS Analysis of Chemicals of Environmental Concern and Byproducts during Drinking Water Production
Source: ACS Meas Sci Au. 2026 Jun 4;6(4):1105–15. doi: 10.1021/acsmeasuresciau.6c00061 (PMC13495728; doi:10.1021/acsmeasuresciau.6c00061)
Supplement: Supplementary file 1 [file tg6c00061_si_001.pdf]

Code_markdown

2025-10-10

Data pre-procesing and offline prioritization (code for PI dataset only)

The converted files were processed with the xcms open Bioconductor R package that allows for obtaining detection and retention time alignment for non-targeted feature profiling (i.e. ions characterized by a unique exact mass and retention time).

```
library(xcms)
library(magrittr)
library(ggplot2)
library(faahKO)
library(RColorBrewer)
library(pander)
library(pheatmap)
library(MsExperiment)
library(xlsx)
library(dplyr)

##  ---------------------------------------------------------------------------
----

##how to read and import converted LC-MS files (mzXML MS1 Full Scan - TIC)
XMLfiles<-dir(path = ".", pattern = ".mzXML", full.names = TRUE,
              recursive = TRUE)

## FROM https://bioconductor.org/packages/release/bioc/vignettes/xcms/inst/do
c/xcms.R##

## Create a phenodata data.frame which refers to sample groups of your batch
pd <- data.frame(sample_name = sub(basename(XMLfiles), pattern = ".mzXML",
                                   replacement = "", fixed = TRUE))
## in case of defined sample groups
pd <- data.frame(sample_name = sub(basename(XMLfiles), pattern = ".mzXML",
                                  replacement = "", fixed = TRUE),
                 sample_group = c(rep("ANC_CT", 9), rep("ANC_IN", 9),
                                  rep("ANC_OUT", 9),rep("ANC_ST", 9),
                                  rep("QC", 27)),
                 stringsAsFactors = FALSE)

## LOADING DATA TO XCMS -----------------------------------------------------
---
data <- readMsExperiment(spectraFiles = XMLfiles, sampleData = pd)
data

spectra(data)
```

```
table(fromFile(data))

sampleData(data)

## -----------------------------------------------------------------------------
---
## Get the base peak chromatograms. This reads data from the mzXML files.
bpcs <- chromatogram(data, aggregationFun = "max")
## Define colors for the two groups
group_colors <- paste0(brewer.pal(6, "Set1")[1:6], "60")
names(group_colors) <- c("ANC_CT", "ANC_IN", "ANC_OUT", "ANC_ST", "QC")

## Plot all chromatograms.
par(mar = c(1, 1, 1, 1)) #set plot margins
plot(bpcs)
plot(bpcs, col = group_colors)

## ----data-inspection-chromatogram, message = FALSE------------------------
---
bpi_1 <- bpcs[1, 1]
rtime(bpi_1) |> head()
intensity(bpi_1) |> head()
plot(bpi_1)
## -----------------------------------------------------------------------------
---
## creating the BPC on the subsetted data if needed
data_ss <- filterRt(data, rt = c(1000, 1500))
bpis <- chromatogram(data_ss, aggregationFun = "max")
plot(bpis, col = group_colors)
## ----data-inspection-tic-boxplot
## Get the total ion current by file
tc <- spectra(data) |>
  tic() |>
  split(f = fromFile(data))
boxplot(tc, col = group_colors[sampleData(data)$sample_group],
        ylab = "intensity", main = "Total ion current")

## Define the rt and m/z range of the peak area
rtr <- c(1320, 1440)
mzr <- c(230.2465, 230.2489)
## extract the chromatogram
chr_raw <- chromatogram(data, mz = mzr, rt = rtr)
plot(chr_raw[1,1], col = group_colors)

data[1] |>
  filterRt(rt = rtr) |>
  filterMz(mz = mzr) |>
```

```r
  plot(type = "XIC")

##Chromatographic peak detection (BLANK)##
#if you want to include isotopes
#cwp <- CentWavePredIsoParam(ppm = 10, peakwidth = c(20, 240), noise = 30000,
                     #prefilter = c(6, 100000), mzCenterFun = "wMean",
                     #maxCharge = 1, maxIso = 3)

cwp <- CentWaveParam(ppm = 5, peakwidth = c(3, 60), noise = 40000,
                            prefilter = c(6, 5000), mzCenterFun = "wMean",
                            )
xdata <- findChromPeaks(data, param = cwp)

#inspection
mzr_1 <- 230.2477 + c(-0.005, 0.005)
rtr_1 <- c(1320, 1440)
chr_1 <- chromatogram(xdata[1], mz = mzr_1, rt = rtr_1)
plot(chr_1)

#merge neighboring peaks & inspect
mpp <- MergeNeighboringPeaksParam(expandRt = 4)
xdata_pp <- refineChromPeaks(xdata, mpp)

chr_1 <- chromatogram(xdata_pp[1], mz = mzr_1, rt = rtr_1)
dev.off()
plot(chr_1)

#if we are ok with peak detection move on with some exploration
xdata <- xdata_pp

summary_fun <- function(z)
  c(peak_count = nrow(z), rt = quantile(z[, "rtmax"] - z[, "rtmin"]))

T <- chromPeaks(xdata) |>
  split.data.frame(f = chromPeaks(xdata)[, "sample"]) |>
  lapply(FUN = summary_fun) |>
  do.call(what = rbind)
rownames(T) <- basename(fileNames(xdata))
pandoc.table(
  T,
  caption = paste0("Summary statistics on identified chromatographic",
                   " peaks. Shown are number of identified peaks per",
                   " sample and widths/duration of chromatographic ",
                   "peaks."))

## Extract a list of per-sample peak intensities (in log2 scale)
ints <- split(log2(chromPeaks(xdata)[, "into"]),
              f = chromPeaks(xdata)[, "sample"])
boxplot(ints, varwidth = TRUE, col = group_colors,
```

```r
       ylab = expression(log[2]~intensity), main = "Peak intensities")
grid(nx = NA, ny = NULL)



## Perform ALIGNMENT
xdata <- adjustRtime(xdata, param = ObiwarpParam(binSize = 1))

bpis_adj <- chromatogram(xdata[11:13], aggregationFun = "max", chromPeaks = "
none")
par(mfrow = c(3, 1), mar = c(4.5, 4.2, 1, 0.5))
sample_colors <- group_colors[bpis_adj$sample_group]
plot(bpis_adj, col = group_colors)
plot(bpis_adj)

## correspondance --------------------------------------------------------

## Perform the correspondence

## Define the mz slice.
mzr <- c(293.17, 293.18)

## Extract and plot the chromatograms
chr_mzr <- chromatogram(xdata, mz = mzr)
## Define the parameters for the peak density method
pdp <- PeakDensityParam(sampleGroups = sampleData(xdata)$sample_name,
                        minFraction = 0.6, bw = 30, binSize = 0.5)

plotChromPeakDensity(bpis_adj, param = pdp,
                     peakPch = 16)
xdata <- groupChromPeaks(xdata, param = pdp)

xdata_ppm <- groupChromPeaks(
  dropFeatureDefinitions(xdata),
  PeakDensityParam(sampleGroups = sampleData(xdata)$sample_name,
                   minFraction = 0.7, bw = 30, ppm = 5))

## Calculate m/z width of features
mzw <- featureDefinitions(xdata)$mzmax - featureDefinitions(xdata)$mzmin
mzw_ppm <- featureDefinitions(xdata)$mzmax -
  featureDefinitions(xdata)$mzmin
plot(featureDefinitions(xdata)$mzmed, mzw_ppm,
     xlab = "m/z", ylab = "m/z width", pch = 21,
     col = "#0000ff20", bg = "#0000ff10")
points(featureDefinitions(xdata)$mzmed, mzw, pch = 21,
       col = "#ff000020", bg = "#ff000010")
## gap filling-blank sub-export------------------------------------------
----------
```

```r
#GAP FILLING
xdata <- fillChromPeaks(xdata, param = ChromPeakAreaParam())

featureValues(xdata, value = "into") |> head()


## FEATURE LIST building & export

df_grouped <-as.data.frame((do.call(cbind, xdata@featureDefinitions[,1:6])
                                    ) |> cbind(featureValues(xdata,
                                                             value = "into"))
)

write.xlsx(df_grouped, "FL_withNoisy.xlsx", sheetName = "Sheet1",
           col.names = TRUE, showNA = T
           )

#Feature blank filtering & export


df_sample <- read.xlsx("FL_withNoisy.xlsx", sheetIndex = 1)
df_blank <- read.xlsx("EL_FEB2024POS.xlsx", sheetIndex = 1)

noisy_features <- which(round(df_sample$mzmed, digits = 3) %in%
                          round(df_blank$mzmed, digits = 3))

df_sample_sub <- df_sample[-noisy_features,]


set.seed(342) #random replace NAs
vals <- seq(from = 10000, to = 10100, by = 2)

df_sample_sub[is.na(df_sample_sub)] <- sample(vals, sum(is.na(df_sample_sub))
,
                                              replace = TRUE)

write.xlsx(df_sample_sub, "featurelist_POS.xlsx", row.names=T,
           col.names = T)

## PCA-----------------------------------------------------------------------------
------
#dataset exploration by PCA

library(factoextra)
```

```
df_POS <- read.xlsx("featurelist_POS.xlsx", sheetIndex = 1, row.names = 1)

sample_group = c(rep("ANC_CT", 9), rep("ANC_IN", 9),
                 rep("ANC_OUT", 9),rep("ANC_ST", 9),
                 rep("QC", 27)) #groups for visualization

sample_group = as.data.frame(sample_group)


df_PCA_POS <- cbind(df_POS$mzmed, df_POS[, 8:70])
df_PCA_POS <- t(df_PCA_POS)
df_PCA_POS <- as.data.frame(df_PCA_POS[-1,]) #df for PCA
df_PCA2_POS <- cbind(df_PCA_POS, sample_group) #df conttaining groups



res.pca_POS <- prcomp(df_PCA_POS, scale = TRUE) #Compute PCA

fviz_eig(res.pca_POS, addlabels = TRUE)#Visualize eigenvalues (scree plot EV%
)


fviz_pca_ind(res.pca_POS, axes = c(1, 2), label="none",  habillage = df_PCA2_
POS$sample_group,
             addEllipses=TRUE, ellipse.level=0.95,
) #score(individual) plot

fviz_pca_var(res.pca_POS, axes = c(1, 2),
             label="none",
             col.var = "contrib", # Color by contributions to the PC
             gradient.cols = c("#00AFBB", "#E7B800", "#FC4E07"),
             repel = F     # Avoid text overlapping
) #loading plot

fviz_pca_ind(res.pca_POS, axes = c(1, 2),
             col.ind = "cos2", # Color by the quality of representation
             gradient.cols = c("#00AFBB", "#E7B800", "#FC4E07"),
             repel = F     # Avoid text overlapping
)

#normalization QC-based
df_norm <- as.data.frame(t(df_PCA_POS[37:63,]))

df_norm1 <- subset(df_norm, select = -c(D1_POS_EDTA_QC1_S9.mzXML,
                                        D1_POS_EDTA_QC2_S9.mzXML,
                                        D1_POS_EDTA_QC2_S9.mzXML,
                                        S1_D1_POS_EDTA_QC1.mzXML,
                                        S1_D1_POS_EDTA_QC2.mzXML,
```

```r
                                        S1_D1_POS_EDTA_QC3.mzXML,
                                        S3_D1_POS_EDTA_QC1.mzXML,
                                        S3_D1_POS_EDTA_QC2.mzXML,
                                        S3_D1_POS_EDTA_QC3.mzXML
                                        ))

df_norm2 <- subset(df_norm, select = c(D1_POS_EDTA_QC1_S9.mzXML,
                                       D1_POS_EDTA_QC2_S9.mzXML,
                                       D1_POS_EDTA_QC2_S9.mzXML,
                                       S1_D1_POS_EDTA_QC1.mzXML,
                                       S1_D1_POS_EDTA_QC2.mzXML,
                                       S1_D1_POS_EDTA_QC3.mzXML,
                                       S3_D1_POS_EDTA_QC1.mzXML,
                                       S3_D1_POS_EDTA_QC2.mzXML,
                                       S3_D1_POS_EDTA_QC3.mzXML))
v1 <- rowMeans(df_norm1) #as.data.frame(rowMeans(df_norm1))
v2 <- rowMeans(df_norm2) #as.data.frame(rowMeans(df_norm2))
v_Norm <- (v2/v1)

df_POS[ , c(16,25,34,43,47,51,55,9,18,27,36,59,60,61,8,17,26,35,56,57,58) ]
<-
  df_POS[ , c(16,25,34,43,47,51,55,9,18,27,36,59,60,61,
              8,17,26,35,56,57,58)]/v_Norm




df_PCA_norm_POS <- cbind(df_POS$mzmed, df_POS[, 8:70])
df_PCA_norm_POS <- t(df_PCA_norm_POS)
df_PCA_norm_POS <- as.data.frame(df_PCA_norm_POS[-1,]) #df for PCA




res.pca_norm_POS <- prcomp(df_PCA_norm_POS, scale = TRUE) #Compute PCA

fviz_pca_ind(res.pca_norm_POS, axes = c(1, 2), label="none",
             habillage = df_PCA2$sample_group,
             addEllipses=TRUE, ellipse.level=0.95,
) #score(individual) plot ---> mi dà errore con df_PCA2
## write -------------------------------------------------------------
---------



write.xlsx(df_POS, "featurelist_POS_norm.xlsx",
           sheetName = "Sheet1")



## EX CODICE ---------------------------------------------------------
-------------
```

```
#PLS-DA-VIP - selection of most relevant components in water classes
library(mdatools)

df_PLSDA <- cbind(df_POS$mzmed, df_POS[, 8:70])
df_PLSDA<- t(df_PLSDA)
df_PLSDA <- as.data.frame(df_PLSDA[-1,]) #df for PCA
df_PLSDA <- cbind(df_PLSDA, sample_group) #df conttaining groups

m.all = plsda(df_PLSDA[,-4360],  df_PLSDA$sample_group, 4, cv = 1,
              cv.scope = "global")
plotRMSE(m.all)
plotXYScores(m.all)
plotPredictions(m.all)

plotVIPScores(m.all, ncomp = 2, type = "h", show.labels = F)
vip = as.data.frame(vipscores(m.all, ncomp = 2))
write.xlsx(vip, "PLSDA_VIP_featurelist_POS.xlsx",
           sheetName = "Sheet1", row.names=F,
           col.names = F)






## recap ------------------------------------------------------------------
---------

#file excel "featurelist_POS_norm.xlsx"--> 4359 feature
# 4 campioni + 3 QC per ogni data ( 9 date )--> (4+3)*9= 63 campioni
#questo file è l'oggetto df_pos
df_POS<- read.xlsx("featurelist_POS_norm.xlsx", sheetIndex = 1)
df_POS<-subset(df_POS, select = -NA..1)
## PLS-DA-VIP  ------------------------------------------------------------
---------------

# no QCs

library(mdatools)

sample_group_noQC = c(rep("ANC_CT", 9), rep("ANC_IN", 9),
                      rep("ANC_OUT", 9),rep("ANC_ST", 9)) #groups for visuali
zation

sample_group_noQC = as.data.frame(sample_group_noQC)
```

```
df_PLSDA<- df_POS[,8:43]
df_PLSDA<- t(df_PLSDA)|>as.data.frame()
df_PLSDA <- cbind(df_PLSDA, sample_group_noQC)

m.all = plsda(df_PLSDA[,-4360],  df_PLSDA$sample_group_noQC, 4, cv = 1,
              cv.scope = "global")

plotRMSE(m.all)
plotXYScores(m.all)
plotPredictions(m.all)
plotVIPScores(m.all, ncomp = 2, type = "h", show.labels = F)

#L vip_noQC = as.data.frame(cbind(vipscores(m.all, ncomp = 2), df_NEG$mzmed))

vip_noQC  = as.data.frame(vipscores(m.all, ncomp = 2))
row.names(vip_noQC)=df_POS$NA.

write.xlsx(vip_noQC, "PLSDA_VIP_featurelist_POS_noQC.xlsx",
           sheetName = "Sheet1")


vip_noQC$ANC_CT <- as.numeric(as.character(vip_noQC$ANC_CT))
vip_noQC$ANC_IN <- as.numeric(as.character(vip_noQC$ANC_IN))
vip_noQC$ANC_OUT <- as.numeric(as.character(vip_noQC$ANC_OUT))
vip_noQC$ANC_ST <- as.numeric(as.character(vip_noQC$ANC_ST))
p1<-vip_noQC[vip_noQC$ANC_CT>1,]
p2<-vip_noQC[vip_noQC$ANC_ST>1,]
p3<-vip_noQC[vip_noQC$ANC_IN>1,]
p4<-vip_noQC[vip_noQC$ANC_OUT>1,]
ora<-c(row.names(p1),row.names(p2),row.names(p3),row.names(p4))|>as.data.fram
e()
colnames(ora)<-"feature"
rm(p1,p2,p3,p4)
p1<-vip_noQC [which(df_POS$NA. %in% ora$feature),]
p2<-df_POS [which(df_POS$NA. %in% ora$feature),]
lista_feature_PLSDAVIP<-cbind(p1,p2)
rm(p1,p2,ora)
lista_feature_PLSDAVIP <- subset(lista_feature_PLSDAVIP, select = -NA.)

colnames(lista_feature_PLSDAVIP)[colnames(lista_feature_PLSDAVIP) == "ANC_OUT
"] <- "VIP_ANC_OUT"
colnames(lista_feature_PLSDAVIP)[colnames(lista_feature_PLSDAVIP) == "ANC_IN"
] <- "VIP_ANC_IN"
colnames(lista_feature_PLSDAVIP)[colnames(lista_feature_PLSDAVIP) == "ANC_ST"
] <- "VIP_ANC_ST"
colnames(lista_feature_PLSDAVIP)[colnames(lista_feature_PLSDAVIP) == "ANC_CT"
] <- "VIP_ANC_CT"
```

```
write.xlsx(lista_feature_PLSDAVIP, "feature_prioritizzate.xlsx",
           sheetName = "PLS-DA-VIP")


## test normality and homogeneity   -----------------------------------------
------------------------------------


library(stats)

norm_test <- df_PLSDA %>%
  group_by(sample_group_noQC) %>%
  summarise_all(.funs = funs(statistic = shapiro.test(.)$statistic,
                             p.value = shapiro.test(.)$p.value))
#data are non-normal!
var_test <- fligner.test(V707~ sample_group_noQC, data = df_PLSDA)
#data have homogeneous variances


## post-hoc test  -----------------------------------------------------------
------------------

library(rstatix)

colnames(df_PLSDA[1:4359]) <- df_POS$NA.


wilcox_test(df_PLSDA, FT0001 ~ sample_group_noQC,
            conf.level = 0.95, p.adjust.method = "BH")

#games_howell_test(df_PLSDA, V1 ~ sample_group_noQC,
#conf.level = 0.95)

# Iterating the test across all features

wilcoxtest_POS <- list()
for(i in (1:4359)){
  wilcoxtest_POS[[i]]<-pairwise.wilcox.test(t(df_PLSDA[,i])[1,],
                                            df_PLSDA$sample_group_noQC,
                                            paired = F,
                                            p.adjust.method = "BH", exact = F
)[[3]]
}

#results and p-values
```

```r
res = lapply(wilcoxtest_POS, function(x) {x[is.na(x)] = 2; x})
res = lapply(res,function(x) x<0.05)
res = lapply(res, sum) # sum considera TRUE come 1 e FALSE come 0
names(res) <-df_POS$NA.
count <- table(unlist(lapply(res, unique)))
count
# count the features with p <0.05

lista_wilcoxtest_POS<-names(res)[res>0]|> as.data.frame() #<0.05 list

colnames(lista_wilcoxtest_POS)<-"Feature significative Wilcoxtest"

lista_wilcoxtest_POS<-df_POS[ which(df_POS$NA. %in%
                                     lista_wilcoxtest_POS$`Feature significa
tive Wilcoxtest`),]


write.xlsx(lista_wilcoxtest_POS, "feature_prioritizzate.xlsx",
           sheetName = "Wilcoxon test",row.names = F,append = TRUE)




##  Comparison wilcoxon & PLS-DA-VIP  --------------------------------------
--------------------------------------



# common features
ora<- df_POS[which(row.names(lista_feature_PLSDAVIP)
                   %in% lista_wilcoxtest_POS$NA.),]



#boxplot:
library(ggplot2)

ora<-read.xlsx('C:/Users/LapoPC/Desktop/DAMIANO/Dati ottenuti MS1/POS/feature
list_POS.xlsx', sheetIndex = 1)
ora1<-read.xlsx('C:/Users/LapoPC/Desktop/DAMIANO/Dati ottenuti MS1/POS/featur
elist_POS.xlsx',sheetindex=2)
ora<-ora[,-c(2:5)]
names(ora1)[5]<-'rtmed.m.'
ora<- rbind(ora,ora1)
ora<-as.data.frame(t(ora))
df_sample_sub=ora
ora<-ora[-c(1:10),]

ora1<-ora[1:9,]
```

```r
ora2<-ora[10:18,]
ora3<-ora[19:27,]
ora4<-ora[28:36,]
ora<- rbind(ora2,ora4,ora1,ora3)
rm(ora1,ora2,ora3,ora4)

ora$sample_group_noQC<-c(rep('ANC_IN',9),rep('ANC_ST',9),rep('ANC_CT',9),rep(
'ANC_OUT',9))

# groups order
ordine_gruppi <- c("ANC_IN", "ANC_ST", "ANC_CT", "ANC_OUT")


ora$sample_group_noQC <- factor(ora$sample_group_noQC, levels = ordine_gruppi
)

#feature selection:
df_sample_sub [df_sample_sub$mzmed>549.9005 & df_sample_sub$mzmed<549.9935,]

# Colours definitions
colore_boxplot <- "#FFA500"
colore_media <- "#4CAF50"
colore_sfondo <- "#F5F5F5"
# Boxplot
grph <- ggplot(ora, aes(x = sample_group_noQC, y = FT0208 )) +
  geom_boxplot(fill = colore_boxplot, alpha = 0.7, color = "#333333") +
  stat_summary(fun = mean, geom = "point", shape = 23, size = 3, color = colo
re_media, fill = colore_media) +
  xlab("Samples") +
  ylab("Intensity") +
  scale_y_continuous(limits = c(0, 500000000), expand = c(0, 0), labels = sca
les::scientific) +
  scale_x_discrete(labels = ordine_gruppi) +
  theme_minimal() +
  theme(
    panel.background = element_rect(fill = colore_sfondo),
    plot.background = element_rect(fill = colore_sfondo),
    panel.grid.major = element_line(color = "#DDDDDD"),
    panel.grid.minor = element_blank(),
    axis.text.x = element_text(size = 14),
    axis.text.y = element_text(size = 14),
    axis.title.x = element_text(size = 16),
    axis.title.y = element_text(size = 16)
  )
graph
```

Annotation

Once converted in .mzML, LC–HRMS/MS files were processed for annotating unknown features beyond level 1:

```
library(xcms)
library(magrittr)
library(ggplot2)
library(faahKO)
library(RColorBrewer)
library(pander)
library(pheatmap)
library(MsExperiment)
library(xlsx)
library(dplyr)
library(BiocParallel)
library(Spectra)
library(MSnbase)
library(BiocManager)
library(MsBackendMgf)
library(msPurity)
library(msPurityData)
library(BiocStyle)
library(MsBackendMassbank)
library(RSQLite)
library(MsCoreUtils)
library(plotly)


#------------------------------



MLfiles<-dir(path = "H:/My Drive/DATI PUBLIACQUA - DATASETS POS NEG/DDA data/
mzXML/ANC_POS/ANC_IN", pattern = ".mzML", full.names = TRUE,
             recursive = TRUE)

pd <- data.frame(sample_name =  sub(basename(MLfiles), pattern = ".mzML",
                                    replacement = "", fixed = TRUE),
                 sample_group = rep("ANC_IN", 3), #c(rep("ANC_CT", 3), rep("A
NC_IN", 3),
                 #rep("ANC_OUT", 3), rep("ANC_ST", 3)),
                 stringsAsFactors = FALSE)


data <- readMsExperiment(spectraFiles = MLfiles, sampleData = pd)
data

#---------------------------
```

```r
#library(Spectra)
#library(MsCoreUtils)

## Extract MS2 from your MsExperiment
ms2 <- spectra(data) |> filterMsLevel(2L)

## (Recommended) basic cleaning & harmonization
#ms2 <- ms2 |>
  #normalize(method = "max") |>              # scale intensities to [0,1]
  #removePeaks(t = 0.01) |>                  # remove tiny peaks (1% of basep
eak)
 #clean()                                   # drop 0-length / NA m/z

## Keep polarity/collision energy as metadata if available
spectraVariables(ms2)  # inspect available fields: precursorMz, rtime, polari
ty, collisionEnergy, ...

BiocManager::install("MsBackendMsp")
library(MsBackendMsp)     # or library(MsBackendMgf)

lib_file <- "H:/My Drive/DATI PUBLIACQUA - DATASETS POS NEG/DDA data/mzXML/AN
C_POS/GNPS-SCIEX-LIBRARY.mgf"
#"GNPS-COLLECTIONS-PESTICIDES-POSITIVE.mgf; GNPS-NIH-SMALLMOLECULEPHARMACOLOG
ICALLYACTIVE.mgf"

## Explicitly create backend first, then pass to Spectra()
be <- MsBackendMgf()
be <- backendInitialize(be, files = lib_file)

lib <- Spectra(be)

scores <- compareSpectra(
  x = ms2,                 # your query spectra
  y = lib,                 # GNPS library
  FUN = MsCoreUtils::ndotproduct,
  MAPFUN = joinPeaks,
  tolerance = 0.001,        # fragment mass tolerance (Da)
  ppm = 20,                # precursor tolerance (ppm)
  requirePrecursor = TRUE,
  minMatchedPeaks = 3,
  SIMPLIFY = TRUE
)

best_hit     <- apply(scores, 1, which.max)
best_score   <- apply(scores, 1, max, na.rm = TRUE)
best_matches <- data.frame(
  query_id = seq_along(best_hit),
```

```r
  lib_id   = best_hit,
  score    = best_score,
  lib_name = lib$NAME[best_hit]
)
head(best_matches)

#-----------MetFrag
library(devtools)
install_github("c-ruttkies/MetFragR/metfRag")
library(metfRag)

#
# first define the settings object
#
settingsObject<-list()
#
# set database parameters to select candidates
#
settingsObject[["DatabaseSearchRelativeMassDeviation"]]<-5.0
settingsObject[["FragmentPeakMatchAbsoluteMassDeviation"]]<-0.001
settingsObject[["FragmentPeakMatchRelativeMassDeviation"]]<-5.0
settingsObject[["MetFragDatabaseType"]]<-"PubChem"
#
# the more information about the precurosr is available
# the more precise is the candidate selection
#
settingsObject[["NeutralPrecursorMass"]]<-549.9525
#settingsObject[["NeutralPrecursorMolecularFormula"]]<-"C7H5Cl2FN2O3"
#settingsObject[["PrecursorCompoundIDs"]]<-c("50465", "57010914", "56974741",
"88419651", "23354334")
#
# pre and post-processing filter
#
# define filters to filter unconnected compounds (e.g. salts)
settingsObject[["MetFragPreProcessingCandidateFilter"]]<-c("UnconnectedCompou
ndFilter","IsotopeFilter")
settingsObject[["MetFragPostProcessingCandidateFilter"]]<-c("InChIKeyFilter")
#
# define the peaklist as 2-dimensional matrix
ms2 <- filterMsLevel(data, 2) |>
  filterMz(c(mz_target - mz_tol, mz_target + mz_tol))

ms2_idx <- which.min(abs(rtime(ms2) - rt_apex))
ms2_peak <- ms2[ms2_idx]

settingsObject[["PeakList"]]<-matrix(ms2_peak, ncol=2, byrow=TRUE)
#
# run MetFrag
#
```

```r
scored.candidates<-run.metfrag(settingsObject)
#
# scored.candidates is a data.frame with scores and candidate properties
```
