# Supplementary material for: A Two-Step Prioritization Framework for the Nontargeted LC-HRMS Analysis of Chemicals of Environmental Concern and Byproducts during Drinking Water Production
Source: ACS Meas Sci Au. 2026 Jun 4;6(4):1105–15. doi: 10.1021/acsmeasuresciau.6c00061 (PMC13495728; doi:10.1021/acsmeasuresciau.6c00061)
Supplement: Supplementary file 2 [file tg6c00061_si_002.pdf]

Supplementary materials of the article "A two-step prioritization framework for the non-targeted LC-HRMS analysis of chemicals of environmental concern and by-products during drinking water production".

Lapo Renai[a,b†], Giulia Bonaccorso[b†], Federico Sinigaglia[b], Andrea Ravalli[c], Daniela Santianni[c], Massimo Del Bubba[b*].

[a]*van 't Hoff Institute for Molecular Science, University of Amsterdam, Science Park 904 1098 XH, Amsterdam, the Netherlands*

[b]*Department of Chemistry, University of Florence, Via della Lastruccia 3, 50019, Sesto Fiorentino, Florence, Italy*

[c]*Publiacqua S.p.A., Via di Villamagna 90/c, 50126 Florence, Italy.*

[†] These authors contributed equally to this work.

*Corresponding author: Massimo Del Bubba, University of Florence; e-mail: massimo.delbubba@unifi.it

S1. Training set and DoE optimization

S1.1 CEC training set

The PFAS standard mixture (2 mg/L) were purchased from Wellington Laboratories Inc. (Guelph, ON, Canada, purity ≥ 99%). The analytical standards of PhCs and UV-filters were purchased from Sigma-Aldrich (St. Louis, MO, USA) and individual standard stock solutions (1 mg/mL) were prepared. All reference standard solutions were stored in the dark at –4 °C. Operative working solutions in ultrapure water were prepared for DoE optimization and batch suitability check with the following composition: PFAS 2 μg/L, PhCs (estrogens excluded) 25–100 μg/L, and estrogens 2000 μg/L.

Table S1 – List of the CEC training set (abbreviations in brackets) including CAS number, chemical formula, pH-dependent Log K_{OW}, pKa (most acidic/most basic, T=25 °C), exact mass of the precursor ion, and related ESI polarity and adduct.

Compound name (Abbreviation)	CAS	Formula	LogD	pKa	m/z	ESI
PFAS (Directive EU 2020/2184)						
Perfluorobutanoic acid (PFBA)	375-22-4	C4HF7O2	-1.22	1.07/n.a.	168.9888	$[M-H-CO_2]^-$
Perfluoropentanoic acid (PFPeA)	2706-90-3	C5HF9O2	-0.52	0.34/n.a.	218.9856	$[M-H-CO_2]^-$
Perfluorohexanoic acid (PFHxA)	307-24-4	C6HF11O2	0.18	-0.78/n.a.	268.9824	$[M-H-CO_2]^-$
Perfluoroheptanoic acid (PFHpA)	375-85-9	C7HF1302	0.88	-2.29/n.a.	318.9792	$[M-H-CO_2]^-$
Perfluorooctanoic acid (PFOA)	335-67-1	C8HF15O2	1.58	-4.2/n.a.	368.9760	$[M-H-CO_2]^-$
Perfluorononanoic acid (PFNA)	375-95-1	C9HF17O2	2.28	-6.51/n.a.	418.9728	$[M-H-CO_2]^-$
Perfluorodecanoic acid (PFDA)	335-76-2	C10HF19O2	2.98	-5.2/n.a.	468.9696	$[M-H-CO_2]^-$
Perfluorundecanoic acid (PFUnDA)	2058-94-8	C11HF21O2	3.69	-5.2/n.a.	518.9664	$[M-H-CO_2]^-$
Perfluorododecanoic acid (PFDoDA)	307-55-1	C12HF23O2	4.39	-5.2/n.a.	568.9632	$[M-H-CO_2]^-$
Perfluorobutanesulfonic acid (PFBS)	375-73-5	C4HF9O3S	0.25	-3.31/n.a.	298.9424	$[M-H]^-$
Perfluoropentansulfonic acid (PFPeS)	2706-91-4	C5HF11O3S	0.95	-3.32/n.a.	348.9392	$[M-H]^-$
Perfluorohexansulfonic acid (PFHxS)	355-46-4	C6HF13O3S	1.65	-3.32/n.a.	398.9359	$[M-H]^-$
Perfluoroheptansulfonic acid (PFHpS)	375-92-8	C7HF15O3S	2.35	-3.32/n.a.	448.9328	$[M-H]^-$
Perfluorooctanesulfonic acid (PFOS)	1763-23-1	C8HF17O3S	3.05	-3.32/n.a.	498.9296	$[M-H]^-$
Perfluorononanesulfonic acid (PFNS)	68259-12-1	C9HF19O3S	3.75	-3.24/n.a.	548.9264	$[M-H]^-$
Perfluorodecanesulfonic acid (PFDS)	335-77-3	C10HF21O3S	4.45	-3.24/n.a.	598.9232	$[M-H]^-$
Perfluoroundecanesulfonic acid (PFUnS)	749786-16-1	C11HF23O3S	5.16	-3.24/n.a.	648.9200	$[M-H]^-$
Perfluorododecanesulfonic acid (PFDoS)	79780-39-5	C12HF25O3S	5.86	-3.24/n.a.	698.9168	$[M-H]^-$
Perfluoro-2-methyl-3-oxahexanoic acid (GENX)	62037-80-3	C6H4F11NO3	0.47	0.33/n.a.	302.0039	$[M-H]^-$
2,2,3-trifluoro-3-(1,1,2,2,3,3-hexafluoro-3-trifluoromethoxypropoxy)propionic acid (ADONA)	919005-14-4	C7H2F12O4	1.41	0.41/n.a.	332.9784	$[M-H]^-$
PhCs and UV-filters (European Decisions 2015/2018/2020/2022)[a]						
17-Alpha-ethinylestradiol (EE2)	57-63-6	C20H24O2	3.9	10.33/-1.7	295.1697	$[M-H]^-$
17-Beta-estradiol (E2)	50-28-2	C18H24O2	3.74	10.33/-0.9	271.1697	$[M-H]^-$
Estrone (E1)	53-16-7	C18H22O2	4.31	10.33/n.a.	269.154	$[M-H]^-$
Amoxicillin (AMX)	26787-78-0	C16H19N3O5S	-2.49	3.23/7.22	366.1124	$[M+H]^+$
Ciprofloxacin (CIP)	85721-33-1	C17H18FN3O3	-0.77	5.56/8.77	332.1411	$[M+H]^+$
Erythromycin (ERY)	114-07-8	C37H67NO13	0.6	12.45/9	734.4691	$[M+H]^+$
Clarithromycin (CLA)	81103-11-9	C38H69NO13	1.24	12.46/9	748.4848	$[M+H]^+$
Ofloxacin (OFL)	82419-36-1	C18H20FN3O4	-0.80	5.17/8.39	362.15168	$[M+H]^+$
Clindamycin (CLD)	18323-44-9	C18H33ClN2O5S	0.38	12.41/7.55	425.18777	$[M+H]^+$
Azithromycin (AZI)	83905-01-5	C38H72N2O12	-3.05	12.43/9.57	749.5164	$[M+H]^+$

Sulfamethoxazole (SMX)	723-46-6	C10H11N3O3S	0.03	6.16/1.97	254.06	$[M+H]^+$
Trimethoprim (TMP)	738-70-5	C14H18N4O3	0.92	n.a./7.16	291.1458	$[M+H]^+$
Clotrimazole (CLZ)	23593-75-1	C22H17ClN2	5.78	n.a./6.26	345.1159	$[M+H]^+$
Fluconazole (FLZ)	86386-73-4	C13H12F2N6O	0.56	12.68/2.3	307.112	$[M+H]^+$
Miconazole (MCL)	22916-47-8	C18H14Cl4N2O	5.87	n.a./6.48	416.991	$[M+H]^+$
Metaflumizone (MFL)	139968-49-3	C24H16F6N4O2	7.38	11.17/-0.2	507.1256	$[M+H]^+$
Diclofenac (DIC)	15307-86-5	C14H11Cl2NO2	1.37	4/n.a.	294.0088	$[M+H]^+$
Venlafaxine (VEN)	93413-69-5	C17H27NO2	0.74	14.42/8.91	278.2121	$[M+H]^+$
O-desmethylvenlafaxine (O-VEN)	93413-62-8	C16H25NO2	0.59	10.11/8.87	264.1964	$[M+H]^+$
Octocrylene (OCT)	6197-30-4	C24H27NO2	6.78	n.a.	362.212079	$[M+H]^+$
Benzophenone-3 (oxybenzone) (OBZ)	131-57-7	C14H12O3	3.59	8.07/n.a.	229.086544	$[M+H]^+$
Avobenzone (AVB)	70356-09-1	C13H18O2	3.94	n.a.	207.138579	$[M+H]^+$

[a]*Decisions (EU) 2015/495, 2018/840, 2020/1161, and 2022/679.*

S1.2 Study samples

Table S2 – Sampling points involved in the study design along the drinking water production chain, the abbreviation used throughout the text, and the description of the treatment stages.

**Sample type**	**Abbreviation**	**Treatment details**
Anconella DWTP inlet	ANC_IN	Arno River after mobile and fixed barriers, and mechanical screens
Effluent from the sand filtration stage	ANC_SF	Sand filter outlet, post-disinfected with both sodium hypochlorite and chlorine dioxide. The sand filter treats water previously undergone to pre-disinfection with chlorine dioxide, flocculant addition, and sedimentation.
Effluent from the activated carbon filtration stage	ANC_CF	The effluent from sand filters, post-disinfected with both sodium hypochlorite and chlorine dioxide, is then treated with granular activated carbon (GAC).
Effluent from the DWTP	ANC_OUT	The effluent from GAC filters is undergone to final disinfection with chlorine dioxide before the water is pumped into the municipal aqueduct.

S1.3 LC-HRMS sensitivity optimization by Design of Experiment

Target for each CMAs was set as the maximization of the chromatographic area for the CEC training set. CMPs were investigated within the following ranges: FoA 0.02–0.5%, FoNH4 0.02–0.5 mM, IV 2–4 kV, and IT 250–450 °C.

$$y_i = \sum_i^n b_i x_i + \sum_{i,j}^n b_{ij} x_i x_j + \sum_i^n b_i^2 x_i^2 + b_0 + \varepsilon \quad \text{Eq. S1}$$

$$Y = XB + E \quad \text{Eq. S2}$$

$$t_a = X w_a \quad \text{Eq. S3}$$

$$u_a = Y c_a \quad \text{Eq. S4}$$

$$maximize\ Cov(X w_a, Y c_a) \quad \text{Eq. S5}$$

Table S3 – D-optimal design matrix obtained for the multilevel (n=3) investigation of CMPs (*power*=45, *I-optimality*=16.18) including the number of the experiments (Exp), the run order, and the selected CMAs.

Exp	Run Order	FoA	FoNH4	IV	IT	PFOA	PFOS	PFBA	PFBS	PFDoDA	MCL	AMX	PFDoS	CIP
N1	10	0.5	10	2	250	3.19E+06	5.65E+07	4.13E+05	1.05E+07	6.18E+06	6.91E+08	1.05E+07	1.95E+06	9.08E+08
N2	16	0.1	10	2	250	1.76E+06	3.82E+07	1.03E+06	6.92E+06	3.98E+06	4.21E+08	8.65E+06	4.74E+05	7.05E+08
N3	12	0.02	2	2	250	2.58E+06	5.14E+07	1.02E+06	5.82E+06	7.90E+06	6.05E+08	6.74E+06	9.74E+05	1.03E+09
N4	5	0.5	2	2	250	2.93E+06	5.48E+07	3.63E+05	8.77E+06	7.00E+06	6.52E+08	1.03E+07	2.71E+06	8.77E+08
N5	9	0.1	0.4	4	250	1.44E+06	3.23E+07	5.49E+05	5.59E+06	1.37E+06	2.83E+08	2.05E+07	2.92E+05	7.40E+08
N6	20	0.02	10	4	250	2.66E+06	4.68E+07	5.67E+05	4.02E+06	6.48E+06	4.39E+08	5.35E+06	1.20E+06	8.25E+08
N7	17	0.5	10	4	250	807000	1.45E+07	1.17E+05	2.53E+06	1.22E+06	2.89E+08	8.48E+06	4.81E+05	7.87E+08
N8	2	0.5	0.4	3	250	3.92E+06	4.55E+07	1.13E+06	1.74E+07	2.83E+06	2.32E+08	6.82E+06	2.15E+05	7.03E+08
N9	1	0.1	0.4	2	550	979000	2.37E+07	2.96E+05	3.50E+06	4.07E+06	8.70E+08	2.96E+07	5.27E+05	1.84E+09
N10	6	0.02	10	2	550	1.11E+07	1.52E+08	5.11E+05	1.97E+07	8.20E+06	1.88E+09	1.09E+07	8.00E+06	3.58E+09
N11	8	0.5	10	2	550	3.75E+06	7.11E+07	8.46E+05	2.64E+07	7.30E+06	3.69E+05	1.00E-04	8.49E+05	1.93E+06
N12	15	0.5	0.4	4	550	1.04E+06	3.03E+07	7.05E+04	8.39E+06	1.69E+07	1.35E+09	1.59E+07	9.62E+06	1.58E+09
N13	13	0.5	10	4	550	2.42E+06	1.27E+08	2.25E+05	1.98E+07	6.53E+06	7.00E+08	2.81E+06	1.15E+07	6.49E+08
N14	21	0.1	10	4	550	850000	1.75E+07	1.64E+05	2.79E+06	4.89E+06	6.73E+08	2.11E+07	5.09E+05	1.41E+09
N15	14	0.02	2	4	550	4.39E+06	1.43E+08	8.76E+05	4.45E+07	5.01E+06	1.08E+09	7.64E+06	5.45E+06	1.54E+09
N16	11	0.5	2	3	550	2.11E+06	1.21E+08	2.47E+05	1.89E+07	2.74E+06	6.09E+07	3.10E+05	9.21E+06	3.38E+07
N17	7	0.5	0.4	2	400	370000	2.29E+07	9.65E+04	3.05E+06	1.03E+06	6.59E+08	4.35E+06	1.17E+06	6.08E+08
N18	3	0.5	2	4	400	302000	1.97E+07	6.37E+04	2.51E+06	9.25E+05	4.68E+08	3.31E+06	1.10E+06	3.88E+08
N19	22	0.02	0.4	3	400	4.83E+06	1.05E+08	9.87E+05	3.38E+07	4.58E+06	1.00E-04	1.00E-05	2.58E+06	2.12E+06
N20	19	0.1	10	3	400	2.47E+07	1.10E+08	5.30E+06	7.18E+07	8.17E+06	5.03E+08	1.87E+07	4.24E+06	1.21E+09
N21	18	0.1	2	3	400	3.66E+07	3.16E+08	7.60E+06	2.16E+08	6.98E+06	8.05E+08	9.90E+06	9.24E+06	1.21E+09
N22	4	0.1	2	3	400	3.78E+07	4.51E+08	6.40E+06	1.66E+08	2.40E+06	8.32E+08	1.34E+07	1.21E+07	1.29E+09
N23	23	0.1	2	3	400	4.59E+07	5.35E+08	5.11E+06	1.09E+08	2.82E+06	7.97E+08	1.25E+07	1.11E+07	1.33E+09

Table S3 – Continued.

Exp	Run Order	FoA	FoNH4	IV	IT	CLA	AZI	SMX	TMP	FLZ	VEN	DIC	E2	E1
N1	10	0.5	10	2	250	1.20E+09	1.37E+08	8.33E+08	1.82E+09	4.32E+08	2.84E+09	2.63E+07	3.72E+05	1.40E+06
N2	16	0.1	10	2	250	9.61E+08	1.32E+08	7.12E+08	1.60E+09	3.97E+08	2.68E+09	3.55E+07	1.74E+06	6.31E+06
N3	12	0.02	2	2	250	1.10E+09	1.58E+08	6.82E+08	1.85E+09	4.90E+08	2.83E+09	2.42E+07	2.16E+07	6.95E+07
N4	5	0.5	2	2	250	1.14E+09	1.31E+08	7.97E+08	1.77E+09	4.09E+08	2.72E+09	2.65E+07	4.11E+05	1.47E+06
N5	9	0.1	0.4	4	250	8.39E+08	1.50E+08	7.16E+08	1.55E+09	4.22E+08	2.55E+09	3.78E+07	1.67E+06	6.03E+06
N6	20	0.02	10	4	250	8.69E+08	1.10E+08	5.71E+08	1.53E+09	4.13E+08	2.25E+09	2.55E+07	1.97E+07	5.63E+07
N7	17	0.5	10	4	250	1.04E+09	1.14E+08	7.89E+08	1.67E+09	3.99E+08	2.67E+09	9.34E+06	6.40E+04	3.03E+05
N8	2	0.5	0.4	3	250	9.32E+08	9.87E+07	7.20E+08	1.54E+09	3.67E+08	2.40E+09	2.59E+07	3.28E+05	1.09E+06
N9	1	0.1	0.4	2	550	2.80E+09	5.43E+08	1.40E+09	2.52E+09	8.09E+08	5.97E+09	4.22E+07	1.68E+04	1.10E+06
N10	6	0.02	10	2	550	3.89E+09	9.63E+08	1.55E+09	4.07E+09	1.46E+09	7.22E+09	4.94E+07	2.66E+06	1.92E+07
N11	8	0.5	10	2	550	2.55E+04	1.00E-04	6.08E+04	1.00E-04	2.54E+06	3.32E+05	4.03E+07	1.89E+03	4.49E+04
N12	15	0.5	0.4	4	550	2.69E+09	4.51E+08	1.23E+09	2.38E+09	7.89E+08	4.76E+09	1.86E+07	1.00E-04	1.65E+05
N13	13	0.5	10	4	550	1.34E+09	9.02E+08	3.58E+08	2.42E+09	2.58E+08	4.23E+09	1.64E+07	2.39E+04	4.64E+05
N14	21	0.1	10	4	550	2.33E+09	4.46E+08	1.13E+09	2.28E+09	6.68E+08	4.53E+09	4.12E+07	2.42E+04	1.34E+06
N15	14	0.02	2	4	550	2.83E+09	1.08E+09	5.26E+08	2.01E+09	4.67E+08	5.55E+09	8.02E+07	1.35E+06	4.02E+06
N16	11	0.5	2	3	550	2.69E+08	7.71E+07	5.30E+07	3.89E+08	3.70E+07	8.56E+08	1.66E+07	3.12E+04	4.36E+05
N17	7	0.5	0.4	2	400	1.02E+09	5.77E+08	4.34E+08	2.86E+09	2.32E+08	4.11E+09	1.26E+07	2.77E+03	2.51E+05
N18	3	0.5	2	4	400	7.18E+08	4.08E+08	2.93E+08	2.00E+09	1.77E+08	2.94E+09	1.22E+07	1.69E+04	2.97E+05
N19	22	0.02	0.4	3	400	1.00E-04	1.00E-04	1.00E-04	1.00E-04	1.00E-04	2.96E+05	6.09E+07	3.78E+06	1.41E+07
N20	19	0.1	10	3	400	1.76E+09	2.78E+08	1.27E+09	2.56E+09	6.71E+08	4.10E+09	4.67E+07	2.31E+05	1.18E+06
N21	18	0.1	2	3	400	1.69E+09	3.51E+08	9.81E+08	2.52E+09	5.52E+08	3.88E+09	3.08E+07	1.35E+05	1.84E+06
N22	4	0.1	2	3	400	1.76E+09	3.62E+08	1.04E+09	2.53E+09	6.67E+08	4.01E+09	2.95E+07	1.44E+05	4.69E+06
N23	23	0.1	2	3	400	1.81E+09	3.59E+08	1.03E+09	2.48E+09	6.76E+08	3.90E+09	2.70E+07	1.26E+05	4.25E+06

Table S4 – Summary of fit of the quadratic modelling for the selected CMAs including quality in fitting (R^2) and in prediction (Q^2), model validity, reproducibility, and model significance in fitting and prediction by ANOVA (*P*, <0.05 in bold).

CMAs	R^2	Q^2	Model Validity	Reproducibility	*P*-value
PFOA	0.704	0.566	0.592	0.861	**0.04**/0.196
PFOS	0.683	0.379	0.459	0.930	**0.015**/0.115
PFBA	0.816	0.668	0.291	0.980	**<0.01**/.0.059
PFBS	0.750	0.580	0.514	0.935	**<0.01**/0.144
PFDoDA	0.358	0.082	0.814	0.417	**0.05**/0.476
MCL	0.545	-0.150	-0.200	0.998	0.108/**<0.01**
AMX	0.657	0.232	0.437	0.940	**<0.01**/0.105
PFDoS	0.501	0.290	0.503	0.885	**0.05**/0.137
CIP	0.574	0.325	-0.138	0.993	**<0.01/0.01**
CLA	0.609	0.387	-0.200	0.996	**<0.01/<0.01**
AZI	0.493	0.284	-0.200	1.000	**<0.01/<0.01**
SMX	0.620	0.213	-0.200	0.995	**0.04/<0.01**
TMP	0.447	-0.051	-0.200	0.999	0.273/**<0.01**
FLZ	0.620	0.137	0.321	0.952	**0.04**/0.07
VEN	0.619	0.262	-0.200	0.998	**0.02/<0.01**
DIC	0.791	0.623	0.220	0.984	**<0.01/0.04**
E2	0.565	0.209	-0.200	1.000	**0.02/<0.01**
E1	0.787	0.667	0.653	0.922	**<0.01**/0.25

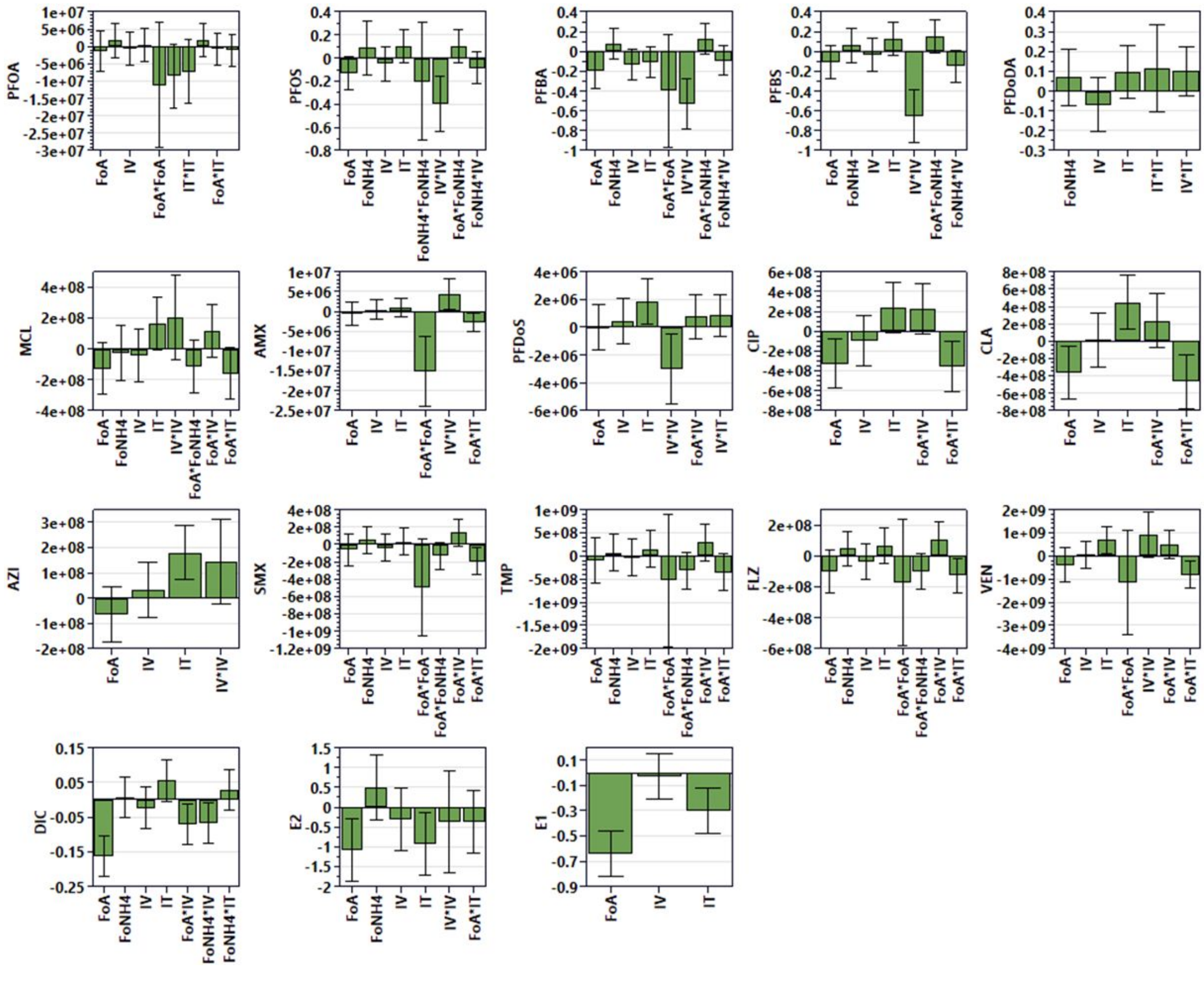

Figure S1 – Graphical analysis of the coefficients of the CMPs (FoA, FoNH4, IV, and IT) used to obtain the quadratic models of the 18 CMAs investigated within the response surface methodology phase.

The chromatographic areas of PFOS, PFBA, PFBS and PFDoS were significantly affected by the IV quadratic term, indicating an increase in sensitivity as IV increases. However, the same CMAs were characterized by a negative influence of FoA, being addressable to the more probable ionic coupling of $[M–H]^-$ and $[M–H–CO_2]^-$ PFAS precursors with H^+ ions in presence of high eluent acidity. FoA linear term also decreased the chromatographic areas of CLA and CIP, even though its interaction with IT positively balanced this contribution, as for increasing IT values the areas were increased. Similarly, AZI sensitivity was significantly promoted by IT. The quadratic term of acidity indicates the influence of this parameter on the response of AMX, SMX, TMP, FLZ and VEN, highlighting the possibility of maximizing their signal, through an appropriate selection of the FoA concentration, due to the presence of basic groups in their structure. However, for DIC, E2, and E1 chromatographic areas were significantly reduced by FoA linear term.

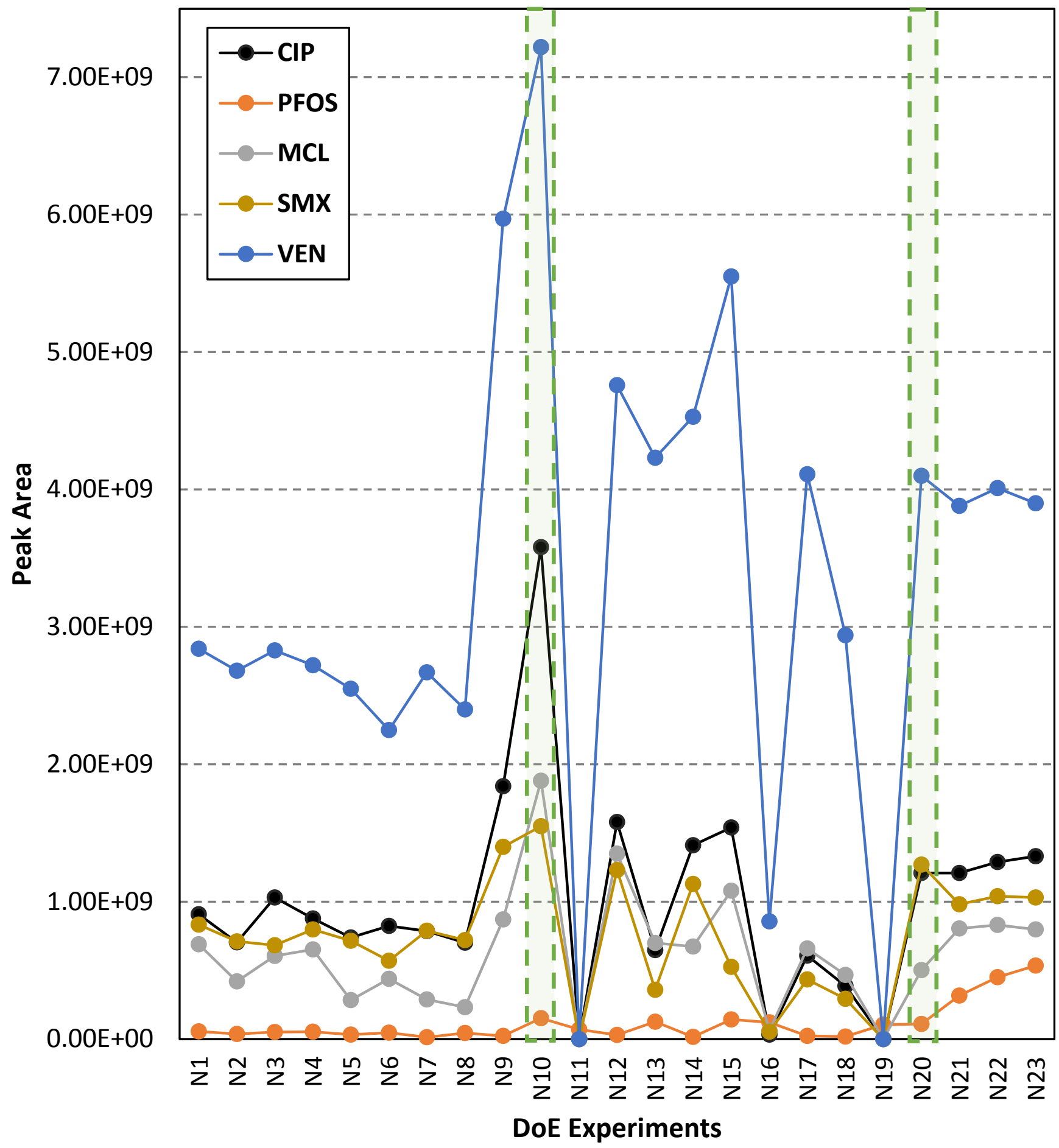

Figure S2 – Chromatographic peak area (CMA) trends for representative chemicals of the training set across the experiments carried out during the DoE. Green-dashed boxes highlight the experiments maximizing the CMA and contributing to the sweet-spot plot and to the selection of final set point.

Table S5 – Ratio between experimental chromatographic areas (mean, n = 3) and the sweet-spot plot predicted values for the selected setpoints. Bold-formatting highlights the ratios exciding the set threshold of 0.75 benchmarking good predictive accuracy. Setpoint 1: FoA – 0.020%, FoNH4 – 10 mM, IV – 2.9 kV, and IT – 469 °C. Setpoint 2: FoA – 0.060%, FoNH4 – 9.6 mM, IV – 2.6 kV, and IT – 353 °C. Setpoint 3: FoA – 0.057%, FoNH4 – 9.7 mM, IV – 2.5 kV, and IT – 348 °C.

CMAs	Setpoint 1	Setpoint 2	Setpoint 3
PFOA	0.14	0.07	0.27
PFOS	0.16	0.13	**0.85**
E2	0.23	0.12	0.10
E1	**1.35**	**0.83**	**0.77**
DIC	**5.17**	**1.31**	**0.91**
PFBA	0.16	0.10	0.30
PFBS	0.33	0.34	0.71
PFDoA	**1.61**	0.67	**1.10**
PFDoS	0.27	0.36	0.74
CLA	0.61	**0.88**	**0.98**
AMX	**1.15**	**0.79**	**0.80**
CIP	**0.94**	**0.95**	**0.83**
AZI	**1.47**	**1.33**	**1.62**
SMX	**0.93**	0.68	0.48
TMP	**1.04**	**0.84**	**0.83**
FLU	**0.94**	0.70	0.45
MCL	**1.16**	**1.20**	**1.33**
VEN	**1.24**	**1.13**	**1.12**
Total	*11*	*9*	*11*

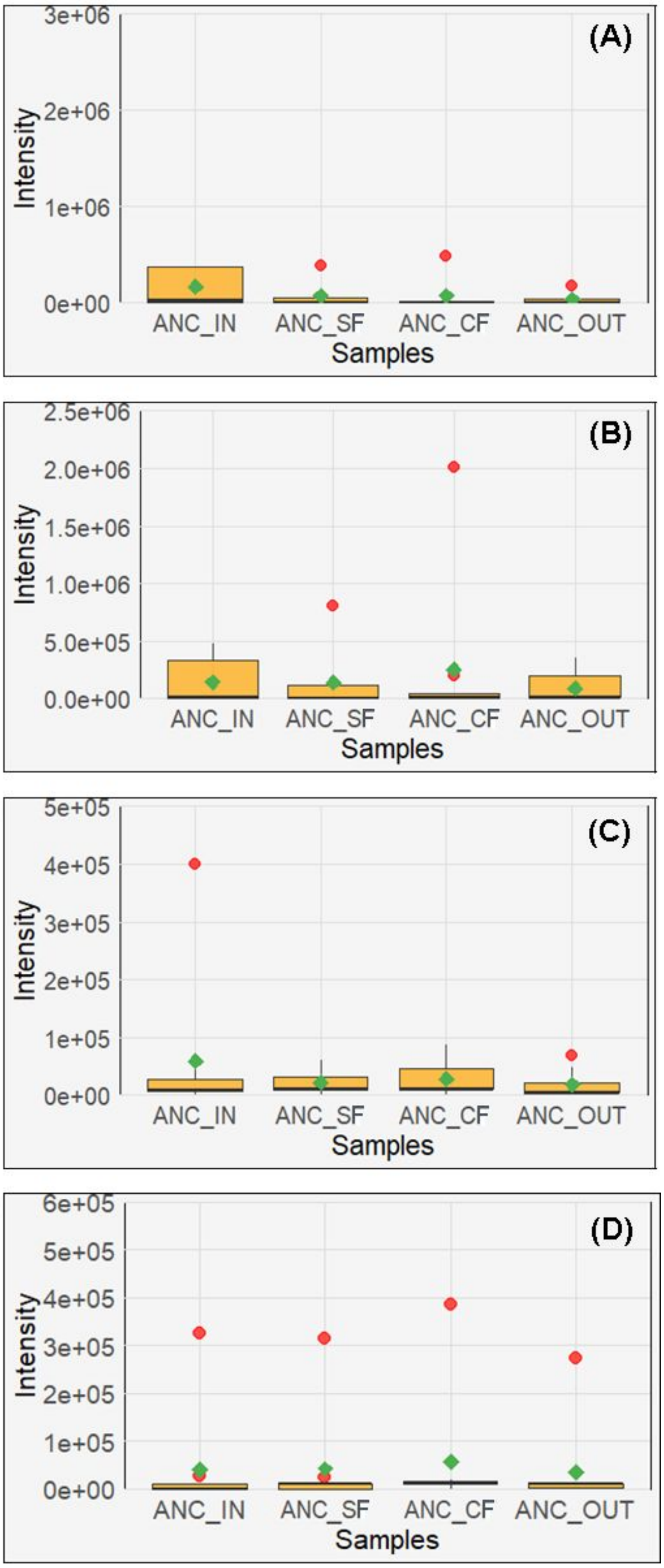

Figure S3 – Box-whiskers plots of level 1 annotated CECs (See Table 1) in the investigated water samples. (A) PFBS (FT0963), (B) PFHpA (FT1287), (C) PFOA (FT1499), and (D) O-VEN (FT1483). The yellow boxes represent the interquartile range (sample size *n*=9), whereas the bold grey line is the median; lower and upper whiskers extend to the maximum or minimum data point within 1.5 box height from the top or bottom of the box; green rhombus is the mean and full red dots are the outliers representing feature transient fluctuations.

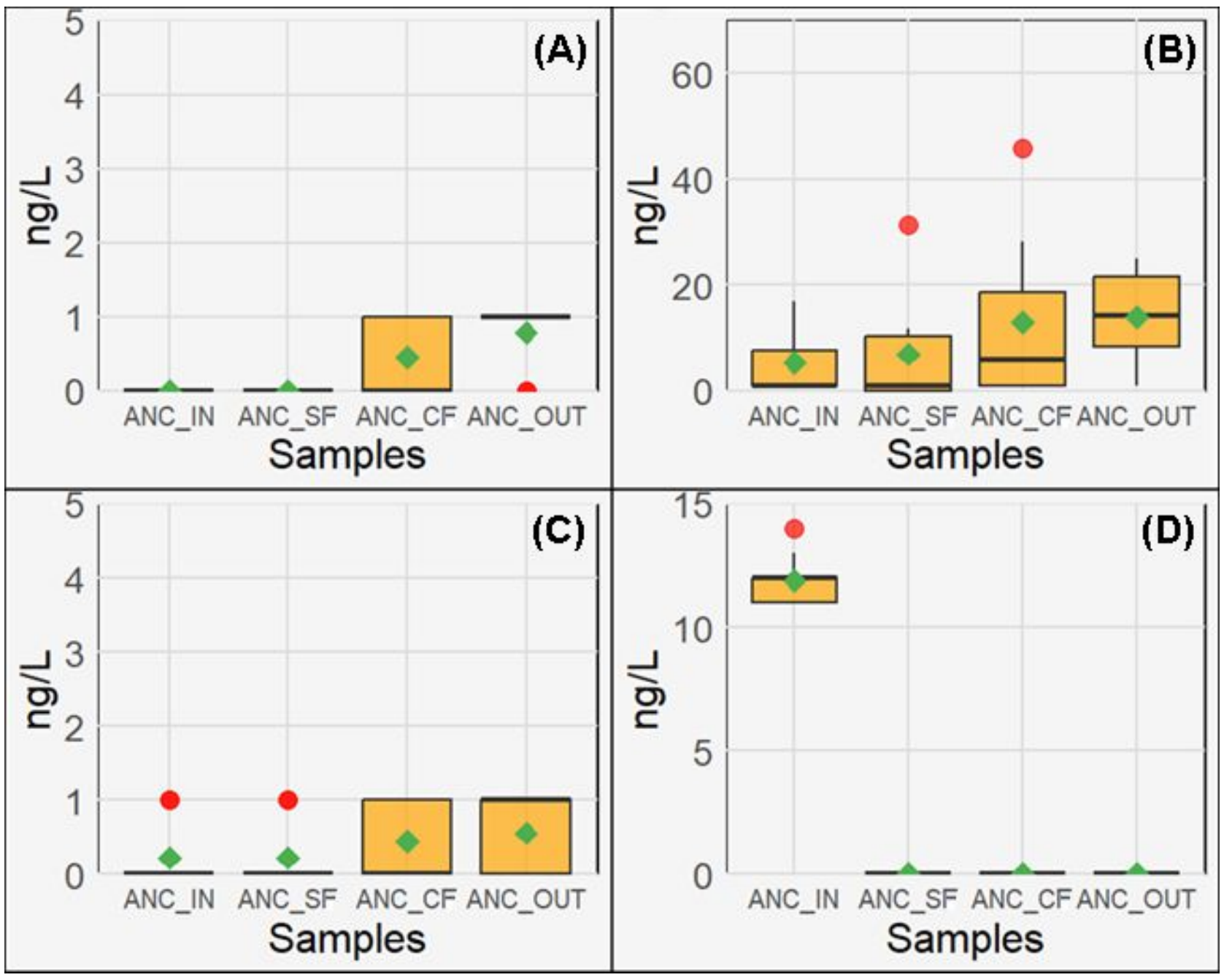

Figure S4 – Box-whiskers plots of the quantification profile for level 1 annotated CECs (See Table 1) in the investigated water samples. (A) PFBS (FT0963), (B) PFHpA (FT1287), (C) PFOA (FT1499), and O-VEN (FT1483).

S2. Data offline prioritization and annotation

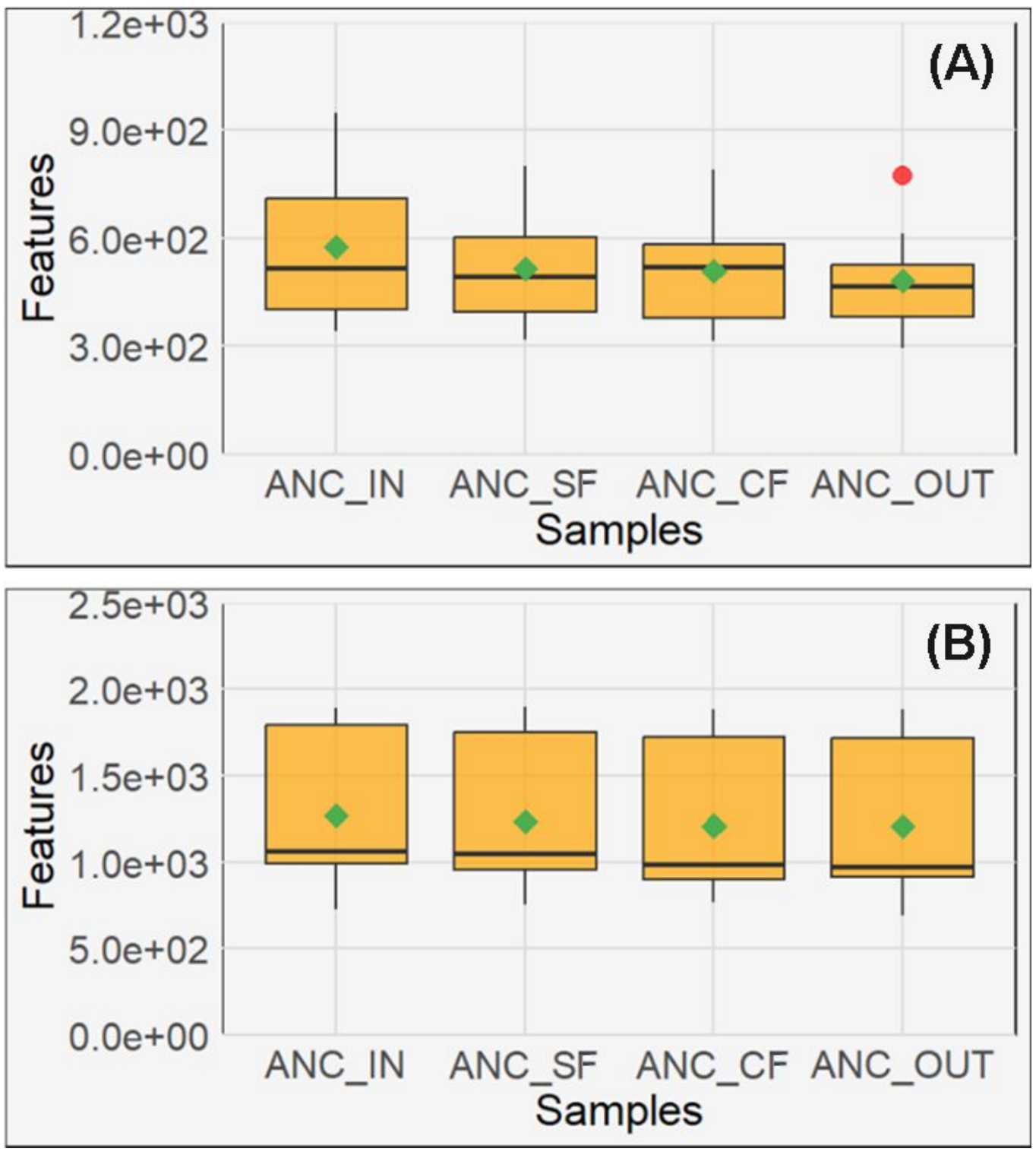

Figure S5 – Box-whiskers plots of the total number of features for each sampling point after data pre-treatment, alignment, and blank subtraction. (A) NI and (B) PI datasets.

Table S6 – Prioritized features for NI and PI datasets according to the workflow reported in section 2.5.2. ID=feature identifier, m/z=exact mass of the precursor ion ($[M-H]^-$ for NI and $[M+H]^+$ for PI), t_R=retention time (min). Features shown in bold are the representative examples reported in Figure 3 of the main text.

PLS-DA-VIP						Wilcoxon					
NI			PI			NI			PI		
ID	m/z	t_R	ID	m/z	t_R	ID	m/z	t_R	ID	m/z	t_R
FT0010	102.9761	3.6	FT1134	229.1199	17.0	FT0308	177.0192	10.2	FT0871	201.1385	10.6
FT0014	102.9623	8.2	**FT1699**	**288.2636**	**23.4**	**FT0417**	**198.0046**	**6.8**	FT1211	237.1021	16.7
FT0032	112.9372	2.2	**FT3326**	**458.1227**	**30.1**	FT0477	212.1371	20.4	FT1308	247.1802	10.9
FT0161	146.9613	21.6	FT5460	861.2477	30.1	FT0573	228.9249	24.9	FT1516	268.1541	9.4
FT0208	158.979	28.8	FT5468	864.2655	31.6	FT0739	258.8392	6.0	FT1881	305.1358	5.7
FT0209	158.979	26.1	FT5760	999.2896	31.7	FT0794	269.1756	18.6	FT1929	311.1333	7.0
FT0257	167.108	15.2				**FT1051**	**317.0912**	**5.4**	FT2006	318.6751	13.8
FT0282	172.0252	2.3				FT1064	319.0109	13.8	FT2100	328.1599	7.0
FT0369	190.9576	4.9				FT1133	332.8998	27.9	FT2116	329.1632	7.0
FT0463	209.1547	21.3				FT1580	435.2964	23.4	FT2321	349.0891	7.0
FT0471	211.0979	15.2				FT1655	455.2647	22.9	FT2327	349.7031	14.4
FT0510	218.0044	14.1				FT1736	480.9699	27.1	FT2412	358.2068	8.9
FT0534	221.8425	6.9				FT2188	647.3464	25.0	FT2413	358.2066	6.8
FT0538	222.158	21.4				FT2221	659.0744	2.0	FT2424	359.21	8.9
FT0557	227.0929	12.7							FT2534	371.2148	14.9
FT0559	227.1289	19.2							FT2562	373.1893	8.4
FT0565	228.0961	12.7							FT2673	385.2065	10.0
FT0586	230.9494	15.3							FT2699	388.2535	8.3
FT0615	237.1085	21.4							FT2772	396.2641	18.7
FT0624	239.1289	21.0							FT2833	403.2363	10.1
FT0625	239.0921	14.4							**FT2930**	**415.2393**	**16.3**
FT0630	239.9898	18.2							FT2945	416.2122	9.6
FT0655	245.1028	11.5							FT2958	417.2155	9.6
FT0671	248.582	24.1							FT3059	428.2851	17.3
FT0685	251.1651	22.5							FT3145	437.7556	16.3
FT0786	267.1611	21.5							FT3221	447.2623	11.1
FT0845	279.1606	19.6							FT3345	459.2575	17.3
FT0863	282.0982	23.6							FT3351	460.2384	10.6
FT0911	291.0835	2.2							FT3371	461.2416	10.6
FT0934	294.9819	16.3							FT3484	474.2535	12.0
FT0935	295.1556	19.8							FT3502	476.3053	10.1
FT0944	296.159	22.5							FT3557	482.8071	17.2
FT0974	301.1662	25.0							FT3632	491.2886	12.0
FT1011	309.1709	21.3							FT3680	498.3782	19.8
FT1013	310.1742	21.3							FT3724	504.2644	11.5
FT1091	325.11	22.0							FT3737	505.2679	11.5
FT1116	329.1529	21.4							FT3787	512.3297	8.3
FT1178	341.161	20.8							FT4029	548.2906	12.3
FT1183	342.1637	20.8							**FT4039**	**549.2938**	**12.3**
FT1186	343.0042	2.2							FT4086	556.2621	10.1
FT1202	345.0466	18.4							FT4240	579.5328	30.2
FT1214	347.1717	25.0							FT4259	582.189	14.6
FT1268	358.738	3.1							FT4310	592.3167	13.1
FT1270	359.1715	19.6							FT4321	593.3201	13.1
**FT1329**	**373.1701**	**19.8**							FT4520	625.6323	30.2
FT1402	389.0513	2.2							FT4574	636.3426	13.7
FT1428	395.244	22.1							FT4597	638.3949	13.9
FT1720	475.132	22.9							FT5084	748.3515	20.3
FT1772	490.2296	20.5							FT5164	770.2842	20.4
FT1801	498.9159	30.4							FT5730	981.2779	30.3
FT2006	587.3594	21.4									
**FT2007**	**588.3628**	**21.4**									
FT2011	589.3654	21.4									
FT2059	603.3542	21.4									
FT2076	609.3409	21.4									
FT2079	610.3443	21.4									
FT2085	611.3471	21.4									
FT2117	623.3074	21.5									
FT2120	625.3141	21.4									
FT2122	626.3176	21.4									
FT2163	636.0857	2.3									
FT2187	647.3076	19.6									
FT2211	656.8855	30.5									

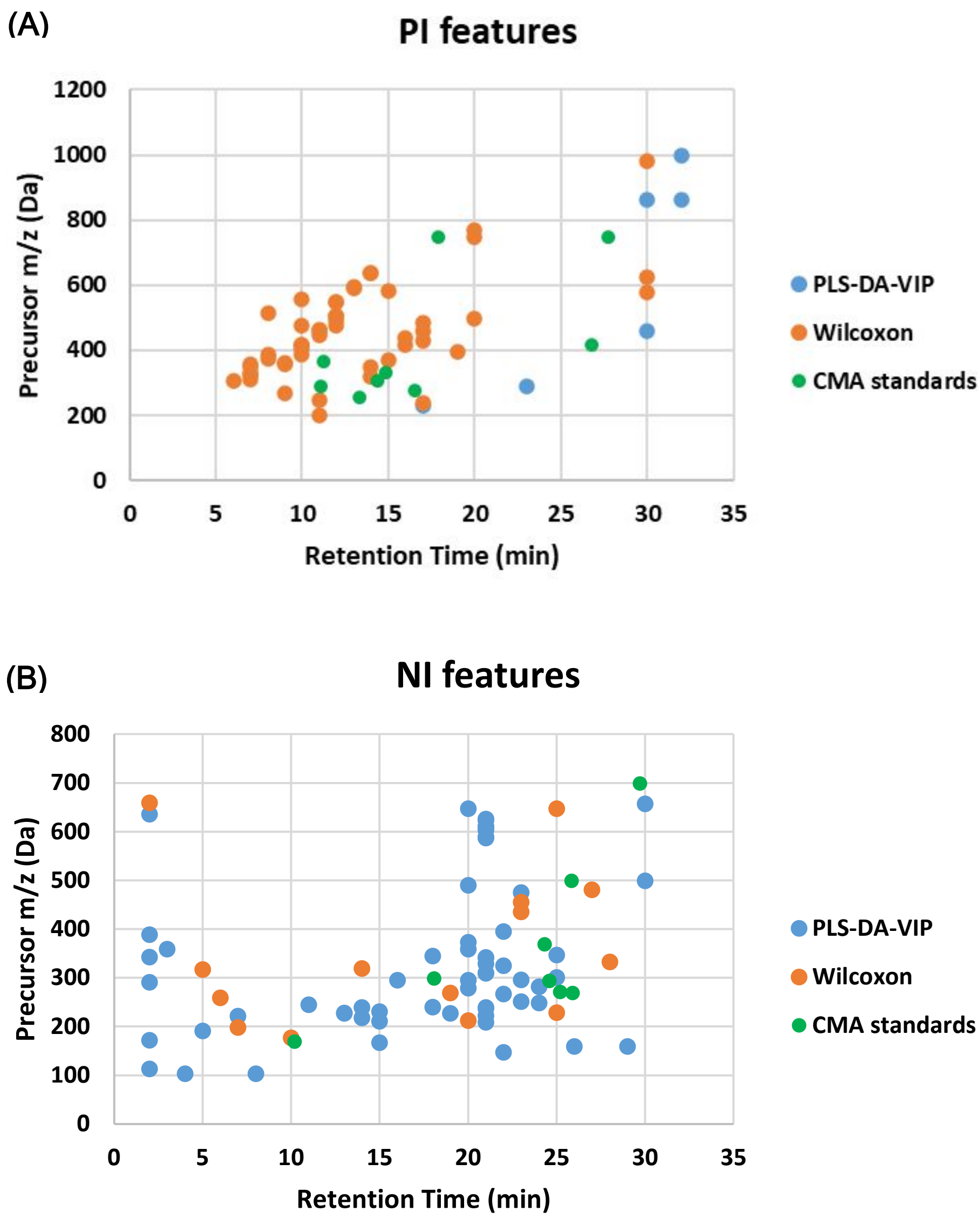

Figure S6 – Retention time vs precursor exact mass (m/z) distribution of the prioritized features and the CMAs belonging to the CEC training set in (A) positive (PI) and (B) negative (NI) ionization polarity datasets.
